# Metagenomic and culture-dependent analysis of *Rhinopithecius bieti* gut microbiota and characterization of a novel genus of *Sphingobacteriaceae*

**DOI:** 10.1038/s41598-024-64727-9

**Published:** 2024-06-15

**Authors:** Qiong Wang, Peng-Chao Zhan, Xiu-Lin Han, Tao Lu

**Affiliations:** 1 Yunnan Institute of Microbiology, Key Laboratory for Southwest Microbial Diversity of the Ministry of Education, School of Life Sciences, Yunnan University, Kunming, Yunnan 650500 PR China; 2 Present Address: Center for Pharmaceutical Sciences, Faculty of Life Science and Technology, Kunming University of Science and Technology, Kunming, Yunnan 650500 PR China

**Keywords:** Metagenome, Carbohydrate active enzyme, *Rhinopithecus bieti*, Polyphasic taxonomy, New genus, Bacteria, Microbial communities

## Abstract

Culture-dependent and metagenomic binning techniques were employed to gain an insight into the diversification of gut bacteria in *Rhinopithecius bieti*, a highly endangered primate endemic to China. Our analyses revealed that *Bacillota_A* and *Bacteroidota* were the dominant phyla. These two phyla species are rich in carbohydrate active enzymes, which could provide nutrients and energy for their own or hosts’ survival under different circumstances. Among the culturable bacteria, one novel bacterium, designated as WQ 2009^T^, formed a distinct branch that had a low similarity to the known species in the family *Sphingobacteriaceae*, based on the phylogenetic analysis of its 16S rRNA gene sequence or phylogenomic analysis. The ANI, dDDH and AAI values between WQ 2009^T^ and its most closely related strains *S. kitahiroshimense* 10C^T^, *S. pakistanense* NCCP-246^T^ and* S. faecium* DSM 11690^T^ were significantly lower than the accepted cut-off values for microbial species delineation. All results demonstrated that WQ 2009^T^ represent a novel genus, for which names *Rhinopithecimicrobium* gen. nov. and *Rhinopithecimicrobium faecis* sp. nov. (Type strain WQ 2009^T^ = CCTCC AA 2021153^T^ = KCTC 82941^T^) are proposed.

## Introduction

Trillions of microorganisms inhabit inside or on the surface of human bodies, and these microbes, especially gut microbes, have a profound impact on human health and well-being^1–3^. Advancements in metagenomics, metabolomics, culturomics, machine learning, and artificial intelligence have shifted the focus of human microbiota research from mere correlation descriptions to causation investigations, enhancing its utility for human well-being^4–6^. Compared with the comprehensive research on human microbiomes, wildlife microbiomes have been less extensively studied. Recent metagenomic analyses by Segal et al. on 180 distinct wild animals across various continents revealed that over 75% of their microbial composition remains uncharacterized. This suggests that wildlife microbiomes possess vast potential as reservoirs for the discovery of novel microbial taxa, genes, enzymes, antimicrobials, and probiotics^7^. Studies on the isolation, identification, and preservation of wildlife microbes not only hold benefits to humans, but also contribute to the conservation of endangered wildlife^8^.

Yunnan snub-nosed monkeys (*Rhinopithecus bieti*) are highly endangered non-human primates endemic to the Southwest of China^9^. They are the only non-human primates that inhabit in harsh conditions at high altitudes (2600–4600 m), with average annual temperatures ranging from 0.9 to 14.3 °C^10–12^. *R. bieti* prefers eating the beard Lichens (*Usnea longissima*), which accounts for 60% to 86% of its annual feeding time^10^. Since the inhabitant microbiota could help their hosts to adapt to specific habitats and diets^13^, it is intriguing to know whether commensal microbes play a role in *R. bieti*’s ability to survive and stay healthy in such a hostile plateau habitat. In addition, many studies have shown that gut microbes in herbivorous animals are rich in carbohydrate-active enzymes, which are used to decompose cell wall polysaccharides (such as cellulose, hemicellulose, pectin and lignin) to provide energy and nutrients for themselves or their symbiotic hosts. Therefore, they are important sources for exploring new carbohydrate-active enzymes for industrial and biotechnological applications. Yet, few studies have been performed on wild primates in this regard^14,15^.

The *Sphingobacteriaceae* family belongs to *Bacteroidetes* phylum that consists of 14 genera. Among them, *Pedobacter*, *Mucilaginibacter* and *Sphingobacterium* are the three largest genera, accounting for about 89% (217/249) of the validly published new species, and *Sphingobacterium* is the type genus of this family^16^. The *Sphingobacteriaceae* family is characterized by the presence of unique sphingolipids in their cell walls and menaquinone 7 as the major respiratory quinone^16^. Members of *Sphingobacteriaceae* live in a variety of environments, such as guts of mammals, soils, fresh waters, wastewaters, composts, active sludges, and rhizosphere^17–23^.

In this study, metagenomic analysis was conducted to elucidate the diversity of gut microbes and the profiles of carbohydrate-active enzymes from *R. bieti*. Culture-dependent analysis was also carried out to investigate the faecal microbes of *R. bieti*, and a series of new species have been isolated and characterized^24,25^. At present, a bacterium designated as WQ 2009^T^ was isolated, and the strain represented a novel genus of the family *Sphingobacteriaceae* based on detailed polyphasic studies.

## Material and methods

### Isolation, cultivation and preservation

Strain WQ 2009^T^ was recovered from the faeces of *R. bieti* collected from the Yunnan Snub-nosed Monkey National Park (27^○^39′N 99^○^21′E; elevation 3000 m), China. The faecal samples of monkeys were collected from their foraging and resting areas. The local temperature during sampling was around 4–12 °C. Samples were taken within 2 h after defecation. Fresh faeces were collected in 15/50 mL sterilized screw centrifuge tubes according to faecal sizes. The faeces were rinsed with sterile saline (0.9%, w/v) for 3 times, then the surface part was removed with sterilized scalpels and the middle part was left for further experiments. Through serial dilutions with 0.1% sterilized Na_4_P_2_O_7_, samples were spread on Columbia Agar plates (Hopebio, China), and were cultured at 30 °C for at least 7 days. The pure culture was obtained as described previously^24,25^ and stored at 4 °C for further study. These pure isolates were cultivated on Columbia or LB agar at 30 °C unless otherwise stated. For long-term storage, bacterial cultures with 20% glycerol (v/v) were maintained at − 80 °C.

### Sample preparation and metagenomic sequencing

Faecal samples of *R. bieti* were collected and handled as described above. For metagenomic sequencing, five tubes of faecal samples were randomly selected and mixed together. The sample was snap-frozen in liquid nitrogen and delivered to QsingKe Biological Technology (Beijing, China) with dry ice for sequencing.

Qualified DNA samples isolated were randomly broken into 350 bp fragments with Covaris ultrasonic breaker (Gene Company Limited, China). The paired-end library was constructed through the steps of DNA ends reparation, 3′-ends A-tailing, adapter ligation, size selection, purification and PCR amplification. The library was qualified by Agilent 2100 Bioanalyzer and ABI StepOnePlus Real-Time PCR System. The qualified library was then sequenced by using Novaseq 6000 sequencing Illumina PE150 platform.

### Metagenome assembly and analysis

The generated raw metagenomes were firstly processed with fastp v0.20.1^26^ (parameter: -q 20 -u 40 -l 15) to eliminate the adapter and low-quality reads. The clean data was assembled and analyzed with SOAP denovo v2.21^27^ with options “-d 1, -M 3, -R, -u, -F -K 55”. The resulting contigs were used for binning through MetaBAT2^28^ (default settings). CheckM v1.2.2 (lineage specific workflow) was applied to the resulting bins, and only those with sufficient quality (≥ 50% completeness, ≤ 10% contamination) were enrolled for de-duplication using dRep v2.5.4^29^ (parameter: -pa 0.90 -sa 0.95 -comp 50 -con 10 -cm larger). Clean reads were mapped to the resulting bins using Bowtie2 v2.4.1^30^ (default settings), and the mapping output was used to estimate the abundance of these bins in the sample through CoverM (parameter: -m tpm) (https://github.com/wwood/CoverM). Taxonomic affiliation of the bins was performed using GTDB-Tk v2.1.0^31^ (classify_wf workflow) based on the Genome Taxonomy Database.

### Construction and functional annotation of the gene catalog of the *R. bieti*

MetaGeneMark v2.10 was used to predict the Open Reading Frames (ORF) from the assembled contigs (≥ 500 bp) obtained above, of which less than 100 nt were filtered out. CD-HIT v4.5.8^32^ (parameter: -c 0.95, -G 0, -aS 0.9, -g 1, -d 0) was used to remove the redundant ORFs. Briefly, the ORFs were clustered at 95% identity and 90% coverage, and the longest sequence was selected as the representative gene sequence to create a non-redundant initial gene catalogue. Genes encoding the carbohydrate-active enzymes (CAZymes) were identified and classified based on the CAZymes database by using the carbohydrate-active enzyme analysis toolkit^33^ (parameter: E-value = 1 × 10^–5^). The CAZy results were then analyzed manually to determine the proportion of different CAZymes. DIAMOND^34^ was used to map these genes to the sequences of bacteria, fungi, archaea, and viruses from NR database with E-value ≤ 1 × 10^–5^ by using blastp, and those with the values ≤ minimum e-value × 10 was selected for further analysis. Finally, clean reads were mapped to the non-redundant ORFs using Bowtie2 v2.4.1 (parameter: -end-to-end, -sensitive, -I 200, -X 400), from which ORFs with alignment count less than 2 were filtered out. And the mapping output was used to calculate the abundance of these ORFs in the sample using the following formula:

$${G}_{k}= \frac{{r}_{k}}{{L}_{K}}\cdot  \frac{1}{\sum_{i=1}^{n}\frac{{r}_{i}}{{L}_{i}}}$$

where ${r}_{k}$ is the reads count, that is, the number of reads mapped to the *k* gene, and ${L}_{K}$ is the gene length, that is, the number of nucleotides in the *k* gene. The index n represents the set of all genes determined in the catalog, and *k* is an index indicating a particular gene.

### Morphological, physiological and biochemical characterization

Transmission electron microscope (JEM-2100, JEOL, Japan) was applied to observe the cell morphology and measure the cell size of strain WQ 2009^T^ cultivated on LB agar (Oxoid) at 30 °C for 24 h. The gliding mobility of the cells was determined by using phase-contrast microscopy (Leica DM2000, Wetzlar, Germany). Gram staining was performed by using the classical staining protocols as well as the rapid KOH lysis method^35^.

Experiments for anaerobic growth, growth temperature, pH and NaCl tolerance were conducted as previously described^17^. All the growth tests of WQ 2009^T^ were performed with LB agar or broth medium for at least 7 days. In brief, the growth temperature was assessed at 0, 4, 10, 15, 20, 25, 28, 37, 40, 45, 50 or 55 °C; the pH tolerance was measured at pH 3.0 to 11.0 with one unit increments by using the following buffers (0.1 M citric acid/0.1 M sodium citrate for pH 3.0–5.0, 0.1 M KH_2_PO_4_/0.1 M NaOH for pH 6.0–8.0, 0.1 M NaHCO_3_/0.1 M Na_2_CO_3_ for pH 9.0–10.0, and 0.05 M Na_2_PO_4_/0.1 M NaOH for pH 11.0) for pH adjustment; for salt tolerance, 0, 0.5, 1, 2, 3, 4, 5, 6, 7, 8, 9 or 10% of NaCl (w/v) was used. Whitley A35 anaerobic workstation (West Yorkshire, UK) was used for testing anaerobic growth.

Hydrolysis of casein, cellulose, Tween 20, Tween 60, Tween 80 and starch was carried out according to the methods described in General and Molecular Microbiology^36^. Catalase activity was assessed by observing whether bubbles formed when a drop of 3% H_2_O_2_ was added to freshly cultured cells. API 20NE, API ZYM galleries and Biolog GEN III MicroPlates (bioMérieux) were used for carbon source utilization, and other biochemical and physiological characterization.

Susceptibility to antibiotics^37^ was tested after incubating strain WQ 2009^T^ at 30 °C for 1 day on LB agar discs (Oxoid) that contain the following antibiotics (μg per disc, unless otherwise stated): amikacin (30), ampicillin (10), cefoperazone (30), chloramphenicol (30), ciprofloxacin (5), clindamycin (2), erythromycin (15), gentamicin (10), kanamycin (30), norfloxacin (10), penicillin (10 IU), piperacillin (100), polymyxin B (300 IU) and vancomycin (30).

### Chemotaxonomic analysis

Cells grown on LB agar at 30 °C for 3–7 days were collected and freeze-dried for chemotaxonomic studies. The methyl-estered cellular fatty acids of strain WQ 2009^T^ were extracted from stationary phase cells as recommended by MIDI technical note^38^, and analysed by using Agilent 7890A GC system according to the standard protocols of the Sherlock Microbial Identification System (version 6.1; MIDI database: RTSBA6). Polar lipids were extracted and analysed by using two-dimensional TLC following protocols reported previously^39^. Respiratory quinones were isolated from freeze-dried cells, and analysed by using HPLC (Agilent 1260) as described^40^.

### Phylogenetic and genome analysis

The whole genomic DNA of WQ 2009^T^ was extracted from mid-log phase cells using the method developed by Andreou^41^. For phylogenetic analysis, the 16S rRNA gene sequence was amplified by using primers 27F (5′-AGA GTT TGA TCC TGG CTC AG-3′) and 1492R (5′-GGT TAC CTT GTT ACG ACT T-3′) with Super-Fidelity DNA polymerase (Vazyme, China). The amplified fragments were cloned into the pBM16A T-vector and sequenced by TsingKe Biological Technology (Beijing, China). The 16S rRNA gene sequence was also extracted from the genome data of WQ 2009^T^ using the online ContEst16 tool build-in EzBioCloud^42^. The 16S rRNA gene sequences of the closely related type strains listed in the EzBioCloud database^43^ and List of Prokaryotic Names with Standing in Nomenclature^44^ were retrieved and aligned with ClustalW^45^, and phylogenetic trees were constructed with neighbor-joining (NJ), maximum-parsimony (MP), and maximum-likelihood (ML) methods using a bootstrap test with 1000 replications in MEGA11^46^.

The whole genome of WQ 2009^T^ was sequenced using the Illumina NovaSeq PE150 platform at TSINGKE Bioinformatics Technology Co., Ltd (Beijing, China). Clean data was obtained by removing the low-quality data from the raw data with readfq version 10, and assembled with SOAP denovo 2.04, SPAdes, AbySS, and integrated with CISA software. GeneMarkS^47^ and tRNAscan-SE program were used to retrieve the related coding genes and the transfer RNA genes, respectively. The assembled genome data was annotated with the prokaryotic genome annotation pipeline in NCBI^48^. Carbohydrate-Active enzymes (CAZymes) were annotated by using the online dbCAN2 meta server^49^, which integrates three tools for automated CAZymes annotation: HMMER, DIAMOND and Hotpep. Antibiotic resistant genes and the secondary metabolism gene clusters were analysed with Diamond^50^ and antiSMASH 6.0^51^, respectively. GC content was calculated from the whole genome sequences. The OrthoANI algorithm implemented on the EzBioCloud and the Genome-to-Genome Distance Calculator version 3.0 implemented on the Type (Strain) Genome Server (TYGS) were used to calculate the ANI values and the DNA–DNA hybridization values, respectively^52,53^. Amino acid sequences of coding regions in the genome of studied strains were inferred by using GeneMarksS-2^54^. The average amino acid identity (AAI) percentages for strain WQ 2009^T^ and three most closely related species *S. kitahiroshimense* 10C^T^, *S. pakistanense* NCCP-246^T^, and *S. faecium* DSM 11690^T^ were estimated with online AAI calculator (http://enve-omics.ce.gatech.edu/aai/index) using default settings (minimun length of 0 aa, minimum identity of 20%, minimum score of 0 bits, and minimum alignment of 50).

For comparative genomic analysis, the genomic DNA sequences of the closely related strains with WQ 2009^T^ were obtained from NCBI GenBank Database for phylogenomics analysis. Functional annotation was conducted using PROKKA^55^. OrthoFinder was used to infer the orthologous genes that are to be aligned by Clustal Omega^56^. Gblocks was used to select conserved blocks from the concatenated alignments^57^. Based on the conserved blocks, a maximum-likelihood tree was constructed by using IQ-TREE^58^.

### GenBank accession numbers

The GenBank/EMBL accession number for the partial 16S rRNA gene sequence of WQ 2009^T^ is MZ413266. The Whole Genome Shotgun project of WQ 2009^T^ has been deposited at DDBJ/ENA/GenBank under the accession number JAGKSB000000000.1. The raw metagenomic data has been deposited to the SRA database under the accession of PRJNA884830 (https://www.ncbi.nlm.nih.gov/sra/PRJNA884830).

## Results and discussion

### Metagenome sequence data statistics and gene assembly

Metagenomic sequencing for the fecal sample of *R. bieti* generated about 40,991,588 raw reads and 40,706,352 clean reads after quality filtering. The effective rate of sequencing data was 99.3%, which was eligible for further analysis. Through gene prediction, a total of 210,425 complete ORFs with an average length and a total length of 641.2 bp and 303.4 Mbp were obtained, which accounted for 44.5% of all ORFs. ORFs only containing the start codon or the end codon were 128,487 (27.2%) and 96,620 (20.4%), respectively.

### Overview of the gut microbiota of *R. bieti*

**Figure 1:**
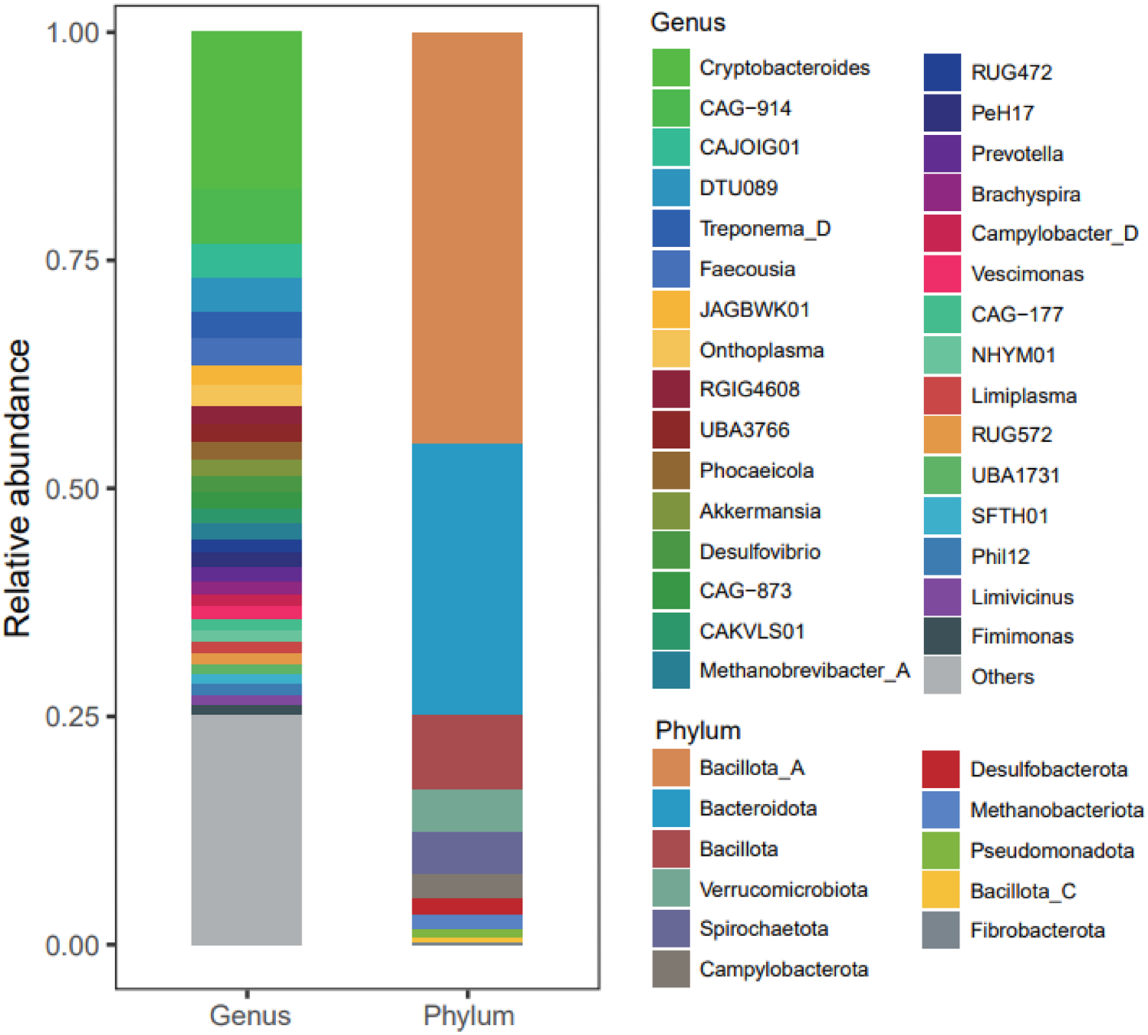
The taxonomic assignment and abundance distribution of MAGs.

The resulting clean data generated 138,262 contigs with lengths > 1000 bp for a total length of 494,930,990 bp and N50 of 6082 bp. The obtained contigs were assembled into 196 bins, and after quality control and de-duplication, 115 bins were left for subsequent analysis. The 115 non-redundant MAGs (metagenome assembled genomes) comprised 72 high-quality MAGs (≥ 80% completeness, ≤ 10% contamination) and 43 medium-quality MAGs (≥ 50% completeness < 80%, ≤ 10% contamination) (Table S1). The taxonomic annotated result showed that almost all the MAGs could be identified at genus and above levels, but only 13.9% (N = 16) could be identified at the species level, which indicated that the intestine of Yunnan snub-nosed monkey contains abundant unknown microbial resources. Among the 115 annotated MAGs (Fig. 1, Table S2), 114 were classified into 10 bacterial phyla, including *Bacillota_A* (N = 69), *Bacteroidota* (N = 21), *Bacillota* (N = 6), *Spirochaetota* (N = 5), *Verrucomicrobiota* (N = 5), *Bacillota*_C (N = 2), *Pseudomonadota* (N = 2), *Bacillota_C* (N = 2), *Fibrobacterota* (N = 1) and *Desulfobacterota* (N = 1) and 1 was identified into archaeal phyla, *Methanobacteriota* (N = 1). Among these, *Bacillota_A* and *Bacteroidota* species were also the two most abundant phyla, accounting for 45.2% and 29.8% of the total abundance, respectively (Table S3). This finding is consistent with previous research conducted in humans and animals, because *Bacillota_A* and *Bacteroidota* species are known for their ability to metabolize polysaccharides, produce short-chain fatty acids and butyrate, maintain intestinal barrier function, and regulate the immune system, which could contribute to their successful colonization of the gut and establishment of an optimal ecological niche^59,60^. Remarkably, only one archaeal strain, *Methanobrevibacter A_smithii*, was detected in the intestine of *R. bieti*, comprising 1.7% of the total abundance. Its significant methane production capacity, coupled with high prevalence, suggests it as the primary methane contributor, offering potential avenues for methane reduction strategies^61^. At the genus level, *Cryptobacteroides* was the most abundant genus, accounting for 17.1% of the total, followed by CAG-914 (6.0%), CAJOIG01 (3.8%), DTU089 (3.6%), Treponema_D (3.0%), Faecousia (2.9%), et cetera (Fig. 1, Table S3). The bacterial diversity revealed in this study was basically consistent with the previous results obtained by Wu et al., who cloned the 16S rRNA genes of the fecal samples and analyzed the bacterial diversity of *R. bieti*^15^. More recently, Xia et al. compared the differences in the composition of gut microbiota between wild foraging and diet-provisioned Yunnan snub-nosed monkeys using 16S rRNA gene and metagenomic functional studies, and the main microbial composition was similar to the results of this study^14^.

### Diversity profile of CAZymes in the microbiota

Microbes play a pivotal role in regulating matter and energy cycles in natural ecosystems, and are an important source of enzymes in biotechnological and industrial applications. Yunnan snub-nosed monkeys feed on the beard Lichens *U. longissimi* as their staple food, supplemented by tender leaves and fruits of other plants. It is interesting to know the potential of their gut microbes to degrade these substances. Based on the experimentally verified and inferred Carbohydrate Active Enzyme (CAZy) database, a total of 18,362 candidates for carbohydrate-active enzyme were predicted from the non-redundant gene catalog. These candidate CAZymes fell into six classes: glycoside hydrolases (GH, 10,167), glycosyl transferases (GT, 4866), carbohydrate-binding modules (CBM, 1935), carbohydrate esterases (CE, 1166), polysaccharide lyases (PL, 220), and auxiliary activities (AA, 8) (Table S4). These CAZymes act synergistically in the breakdown of dietary cellulose, hemicellulose, and pectin to provide energy and nutrients to the gut microbes and their host.

The GH families were the most abundant in the metagenomes, including 112 different families, which accounted for 55.4% of the total CAZymes (Table S4). The top 10 abundant families GH2, GH3, GH43, GH13, GH28, GH78, GH77, GH23, GH94 and GH31 accounted for 45.8% of all the GH enzymes. GH2 and GH3 were the two most abundant families accounting for more than 17% of the total GHs, which have broad activities for the synthesis or degrading of oligosaccharides, such as *β*-galactosidase, *β*-glucosidase, *β*-xylosidase, *β*-manosidase and *α*-l-arabinofuranosidase. GH13 was also one of the most abundant families, which is more specifically involved in the degradation of starch. Other predicted GH enzymes belonged to cellulases, hemicellulases, debranching enzymes and pectin lyases.

The next abundant CAZymes belonged to GT family (the primary enzymes for carbohydrate synthesis), with 50 families accounting for 26.5% of the CAZymes. Among them, GT2, GT4, GT51, GT35, GT28, GT5 and GT26 accounted for 71.9% of GT family enzymes. These enzymes are responsible for catalyzing the transfer of activated nucleotide sugars to carbohydrates during oligosaccharide biosynthesis^62^.

CBM family was the third most abundant CAZymes, including 54 different families, accounting for 10.5% of the CAZymes. Enzymes of CBM family can enhance the catalytic efficiency of GHs by specifically binding to its substrate and increasing the enzyme concentration^63^.

The 220 PLs were distributed in 16 families, which degrade a variety of uronic acid-containing polysaccharides by *β*-elimination mechanism^64^. CEs catalyze the de-*O* or de-*N*-acylation of substituted saccharides and degrade polysaccharides synergistically with GHs^65^. In this study, 1166 enzymes were predicted as CEs and belonged to 13 families. CE4 (356 genes), CE9 (217 genes) and CE12 (164 genes) were the most abundant families, mainly degrading the acetylated pectin, chitin or xylan. Eight genes fell into the AA10 family (formerly CBM33), which are copper-dependent lytic polysaccharide monooxygenases (LPMOs) that act primarily on recalcitrant polysaccharides, such as chitin and cellulose^66^. The enzymes in AA category being much less than those of other families might be due to the fact that AA enzymes are oxidative enzymes and the gut itself is an anaerobic environment.

**Figure 2:**
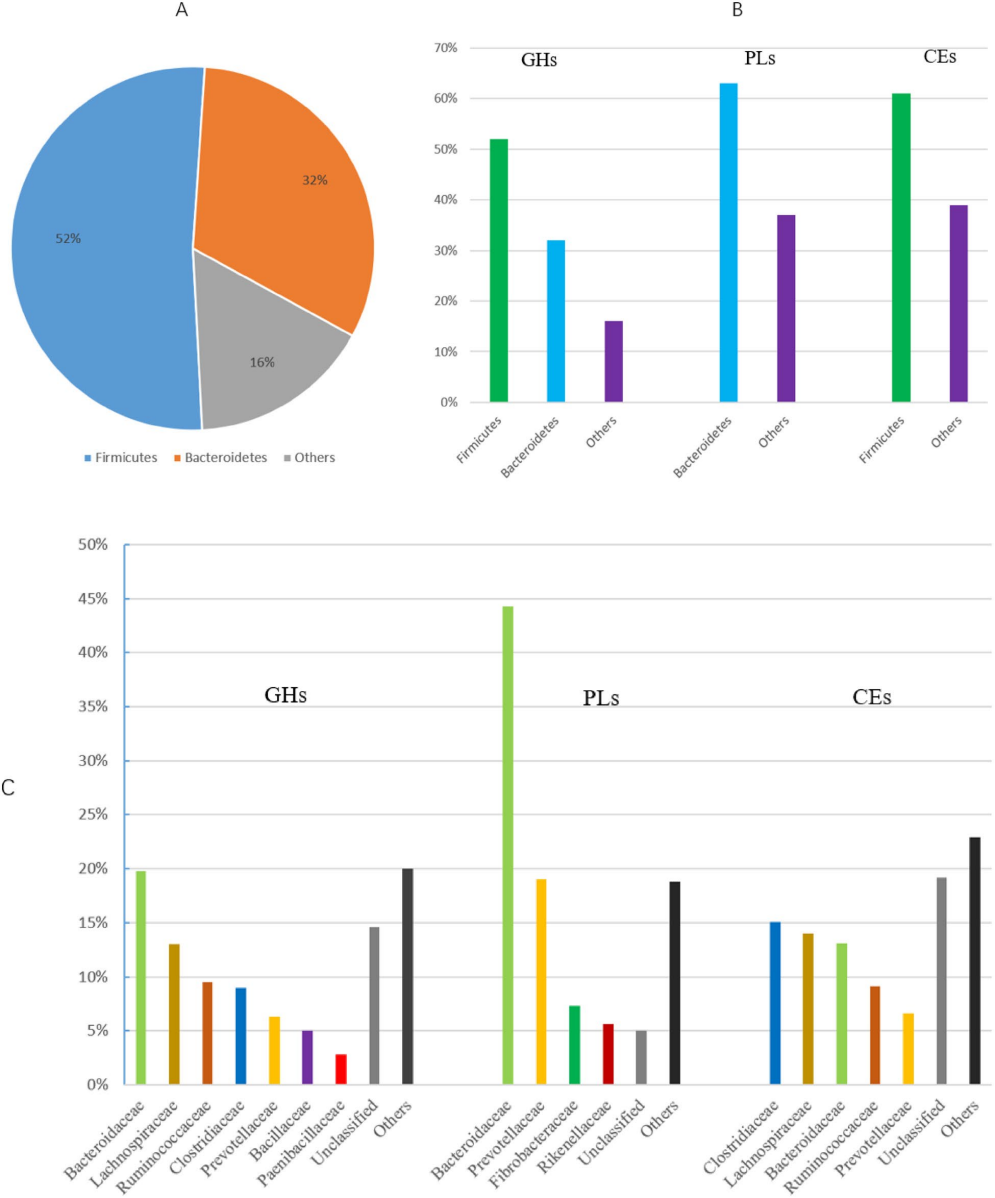
Taxonomic assignments for the genes encoding CAZymes. Phylum-level (**A**) taxonomic assignments for six CAZyme classes GHs, GTs, PLs, CEs, CBMs and AAs; Phylum- (**B**) and family-level (**C**) taxonomic assignments for the genes conding for three CAZyme classes GHs, PLs, and CEs.

Taxonomic profiles of genes encoding CAZymes of GHs, CEs and PLs were also manually analysed. As shown in Fig. 2A, the vast majority (> 84%) of all CAZymes were mainly derived from phylum Firmicutes (52%) and Bacteroidetes (32%). In addition, Firmicutes contributed the most to GHs (52%) and CEs (61%). However, Bacteroidetes was accounted for 32% GHs and 63% PLs (Fig. 2B). Further observations at the levels of family taxonomy (Fig. 2C) revealed that: in the GHs classes, families *Bacteroidaceae*, *Lachnospiraceae*, *Ruminococcaceae*, *Clostridiaceae*, *Prevotellaceae*, *Bacillaceae*, *Paenibacillaceae*, and *Rikenellaceae* contributed more than 65%; in the PLs classes, families *Bacteroidaceae*, *Prevotellaceae* and *Fibrobacteraceae* contributed more than 76%; in the CEs classes, families *Clostridiaceae*, *Lachnospiraceae*, *Bacteroidaceae*, *Ruminococcaceae*, and *Prevotellaceae* contributed about 58%. For GHs and CEs, there were about 15% and 19% unclassified species in the taxonomic level of Family, respectively. The high proportion of unclassified species indicated that many microorganisms with special metabolic potentials have not been discovered in the gut microbiota of *R. bieti*.

### *Bacteria* composition of the gut microbiota in pure culture

A total of 3065 pure strains were randomly picked out from the faecal samples of *R.beiti*. After removing duplicated strains based on the characteristics of morphology, color and colony texture, 412 strains were kept for further study. Through 16S rRNA gene sequencing, sequence alignment and inquiry analysis of these strains, 221 actinomycetes and 191 other bacteria were preliminarily identified. The actinomycetes were distributed in 8 orders, 14 families and 25 genera of the class Actinomycetes, with *Arthrobacter* in *Micrococcaceae* being the most common species (Table S5). The other 191 bacterial strains were distributed in 4 phyla, including Bacteroidetes, Firmicutes, Proteobacteria and Deinococcus-thermus, which were further divided into 7 classes, 10 orders, 19 families and 30 genera, with *Sphingobacterium* having the highest number of strains (Table S6).

From the obtained pure cultures, it was found that the similarities of 16S rRNA gene sequences of 20 strains were less than or equal to 98.7% when compared to those of their most related strains (one of the criteria of new species classification)^67^, indicating that these strains were potential new taxa (Table S7). Among them, WQ 047 and WQ 117 have been identified as new taxon and validly published^24,25^, and renamed as *Sphingobacterium Rhinopitheci* WQ 047^T^ and *Faecalibacter Rhinopitheci* WQ117^T^, respectively. Among the remaining species, WQ 2009^T^ has the lowest sequence similarity with its most similar species. Furthermore, WQ 2009^T^ could not assembled from the faecal metagenome of *R.beiti* based on binning methodology, indicating that it is a part of the rare biosphere. Therefore, we proposed that it belongs to a new genus of *Sphingobacteriaceae* family, and its classification was systematically studied in the following work.

### Cell morphology and physiology

**Figure 3:**
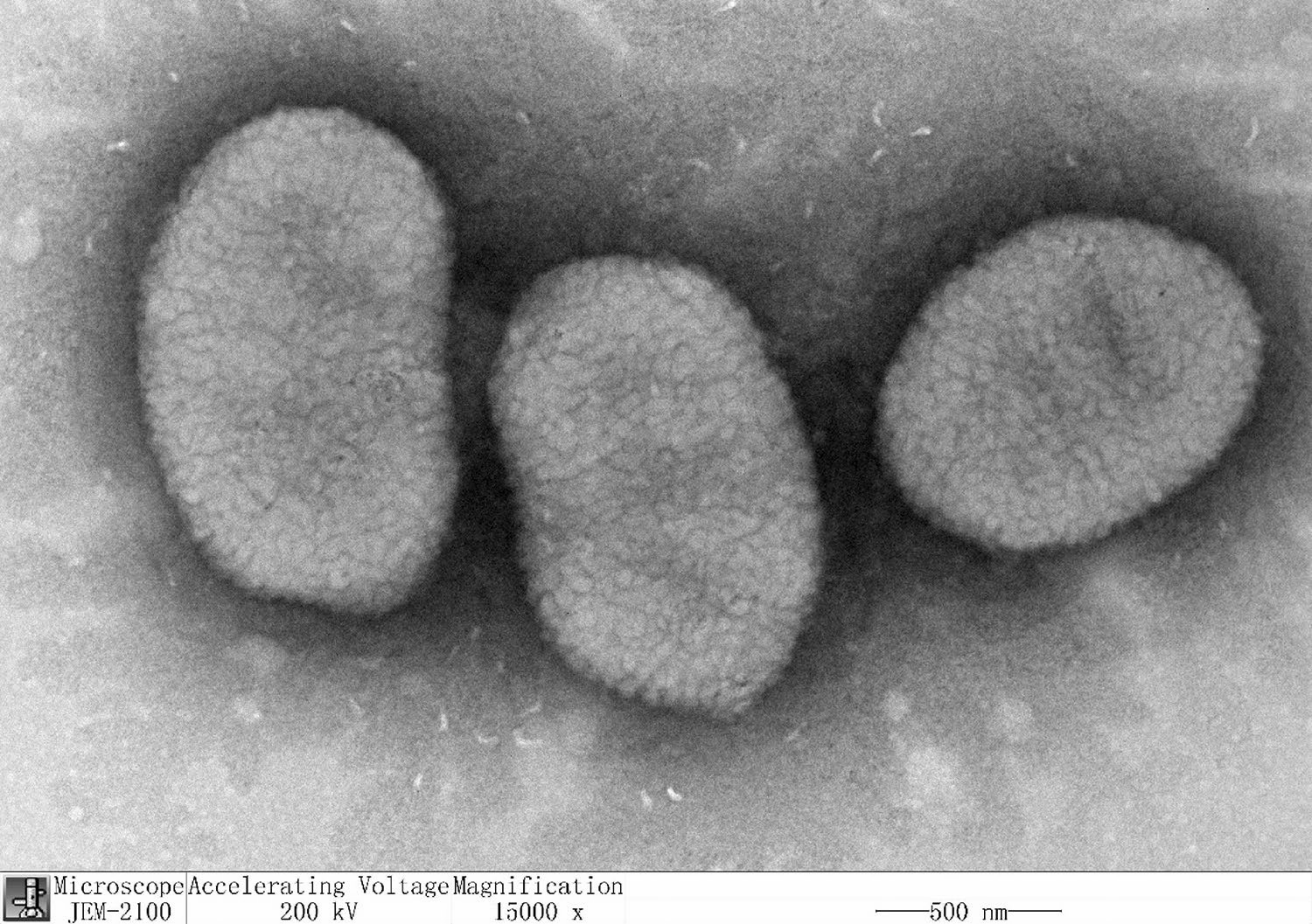
The TEM image of strain WQ 2009^T^ cultured on LB at 30 °C for 1 day. Bar, 500 nm.

The average cell size of strain WQ 2009^T^ was 0.7 ± 0.1 μm in width and 1.1–1.4 μm in length (Fig. 3). The cells were aerobic, Gram-stain-negative, oval-shaped, non-gliding, oxidase and catalase positive, and produced yellow colonies on Columbia Agar. WQ 2009^T^ could grow at 0–35 °C (optimum 20–30 °C), pH 7.0–8.0 (optimum pH 7.0), and with up to 2% (w/v) NaCl (optimum 0–1.5%).

Strain WQ 2009^T^ could hydrolyse esculine, *p*NPG, Tween 20, Tween 40, Tween 60, Tween 80, pectin, starch and cellulose, but not gelatin. The test for H_2_S and indole production was negative. Nitrate was reduced to nitrogen instead of nitrite. WQ 2009^T^ was positive for enzyme activities of alkaline phosphatase, esterase (C_4_), esterase lipase (C_8_), lipase (C_14_), leucine arylamidase, valine arylamidase, cystine arylamidase, trypsin, *α*-chymotrypsin, acid phosphstase, naphthol-AS-BI-phosphohydrolase,* α*-galactosidase,* β*-galactosidase, *α*-glucosidase, *β*-glucosidase, N-acetyl-*β*-glucosaminidase and *α*-mannosidase. Glucose, arabinose, glucosamine and maltose could be assimilated by this isolate. For carbon sources, the strain could use maltose, cellobiose, gentiobiose, turanose, lactose, melibiose, salicin, glucosamine, mannosamine, galactosamine, glucose, mannose, fructose, galactose, fucose, rhamnose, glycerol, glucose-6-PO_4_, frucose-6-PO_4_, glycyl-l-proline, alanine, serine, galactonic acid lactone, glucuronic acid, lactic acid methyl ester, α-keto-glutaric acid, acetoacetic acid and acetic acid as substrates.

Strain WQ 2009^T^ showed resistance to nalidixic acid, aztreonam, amikacin and vancomycin. The cells were susceptible to ampicillin, cefoperazone, chloramphenicol, ciprofloxacin, clindamycin, erythromycin, gentamicin, kanamycin, norfloxacin, penicillin G, piperacillin and polymyxin B. The morphological and physiological characteristics of strain WQ 2009^T^ and its most related species in the family *Sphingobacteriaceae* are summarized in Table 1.

**Table 1:** Differential morphological and physiological characteristics of strain WQ 2009^T^ (1) and its closest relatives: *S. kitahiroshimense* 10C^T^ (2)^73^, *S. pakistanense* NCCP-246^T^ (3)^74^ and* S. faecium* DSM 11690^T^ (4)^75^.

Characteristics	1	2	3	4
Isolation source	Faeces of *R. bieti*	Soil	Rhizosphere	Faeces of *Bos sprunigenius taurus*
Cell size (μm)	0.7 ± 0.1 × 1.1–1.4	0.6–0.8 × 0.5–0.6	1.7–3.3	0.4–0.5 × 0.5–1.0
Cell shape	Oval	Rod	Short rod	Rod
Cell color	Yellow	Yellow or creamy white	Yellowish white	Yellow or creamy white
Motility	No	No	No	No
Gram-stain	Negative	Negative	Negative	Negative
Nitrate reduction	+	−	+	−
Indole production	−	−	−	−
Oxidase	+	+	−	+
Catalase	+	+	+	+
Urease	−	+	+	−
Growth				
Temperature (optimum) (℃)	0–35 (20–30)	4–37 (NA)	16–37 (32)	5–37 (NA)
pH range (optimum)	7.0–8.0 (7.0)	NA	5.0–8.0 (7.0)	NA
NaCl tolerance (optimum) (%, w/v)	0–2.0 (0–1.5)	NA	0–4.0 (0–1.0)	NA
Hydrolysis				
Aesculin	+	+	+	+
Gelatin	−	−	−	−
Tween 40	+	+	NA	NA
Tween 80	+	+	+	NA
Starch	+	NA	+	+
Cellulose	+	NA	NA	−
Pectin	+	NA	NA	−
Substrates				
d-Xylose	−	+	NA	+
d-Glucose	+	+	+	+
l-Arabinose	−	−	+	+
d-Fructose	+	+	+	+
d-Cellobiose	+	+	+	+
Sucrose	−	+	+	+
d-Maltose	+	+	+	+
d-Raffinose	−	−	+	+
l-Rhamnose	+	+	−	−
d-Mannitol	−	−	NA	−
Glycerol	+	−	+	−
Enzyme activity				
Alkaline phosphatase	+	+	+	+
Esterase (C_4_)	+	−	−	NA
Esterase lipase (C_8_)	+	+	+	NA
Lipase (C_14_)	−	−	−	−
Leucine arylamidase	+	+	−	NA
Valine arylamidase	+	+	+	+
Cystine arylamidase	+	−	−	NA
Trypsin	+	−	−	−
α-Chymotrypsin	+	−	−	−
Acid phosphatase	+	+	+	+
Napthol-As-BI-phosphohydrose	+	+	+	NA
α-Galactosidase	+	+	+	+
β-Galactosidase	+	−	−	NA
β-Glucuronidase	+	+	−	−
α-Glucosidase	+	+	+	NA
β-Glucosidase	+	+	+	+
N-acety-β-glucosaminidase	+	+	+	NA
α-Mannosidase	+	+	+	NA
α-Fucosidase	+	−	+	−

‘*S.*’ stands for ‘*Sphingobacterium*’, *NA* no data available, + positive, − negative.

### Chemotaxonomic characterization

As shown in Table 2, the chemotaxonomic properties of strain WQ 2009^T^ were consistent with those of the family *Sphingobacteriaceae*. The major fatty acids were C_15:0_ iso, anteiso-C_15:0_ and Summed Feature 3 (C_16:1_ *ω*7*c*/C_16:1_* ω*6*c*). The main polar lipids were phosphatidylethanolamine (PE), unknown glucosamine phospholipid (APL) and unknown glycolipid (GL) (Fig. S1). The predominant respiratory quinone was MK-7. The chemotaxonomic properties confirm that strain WQ 2009^T^ is affiliated to the family *Sphingobacteriaceae*.

**Table 2:** The cellular fatty acid composition of strain WQ 2009^T^ and its closely related type species.

Fatty acid	1	2	3	4
iso-C_15:0_	31.7	28.9	28.0	43.2
anteiso-C_15:0_	11.4	ND	ND	ND
iso-C_15:0_ 3-OH	2.3	ND	2.7	21.4
iso-C_17:0_ 3-OH	9.4	12.8	ND	ND
C_16:0_	1.6	ND	11.9	7.1
C_16:0_ 3-OH	1.6	ND	4.8	13.2
Summed features 3	19.04	40.3	37.1	48.4

1, WQ 2009^T^; 2, *S. kitahiroshimense* 10C^T^^73^; 3, *S. pakistanense* NCCP-246^T^^74^; 4, *S. faecium* DSM 11690^T^^75^. Values are percentages of total fatty acids. Only those with percentages ≥ 1.0% are presented. ND, not detected or < 1.0%. Fatty acids that cannot be separated by GC using Microbial Identification System (Microbial ID) software are considered to be summed features. Summed feature 3: C_16:1_ *ω7c* and/or C_16:1_ *ω6c*. ‘*S.*’ stands for ‘*Sphingobacterium*’.

### Phylogenetic analysis and genomic characterization

**Figure 4:**
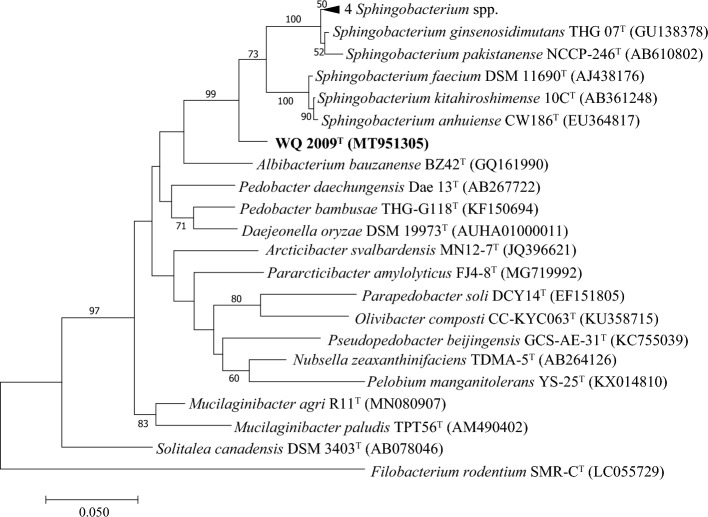
The phylogenetic tree based on 16S rRNA gene sequence of strain WQ 2009^T^ using the maximum-likelihood method. Bootstrap values (expressed as percentages of 1000 replications) of above 50% are shown at branch points. *Filobacterium rodentium* SMR-C^T^ was used as the outgroup. That is, fewer than 2% alignment gaps, missing data, and ambiguous bases were allowed at any position. Bar, 0.05 substitutions per nucleotide position.

The nearly complete 16S rRNA gene sequence (1491 bp) of strain WQ 2009^T^ was determined. Comparative sequence analysis of strain WQ 2009^T^ and the validly published type strains using the EzBioCloud server revealed that the most similar strains were those of the members of family *Sphingobacteriaceae*. WQ 2009^T^ showed the highest 16S rRNA similarity to *S. kitahiroshimense* (94.5%), followed by *S. pakistanense* and *S. faecium* at a 94.3% similarity level (Table 3), which was below or near the recommended threshold of 98.7% and 94.5% for differentiation of a new species and a new genus, respectively^68^. Strain WQ 2009^T^ showed 16S rRNA gene sequence similarities of less than 92.2% to those of the type strains of other 13 genera of family *Sphingobacteriaceae*. To precisely clarify the taxonomic position of WQ 2009^T^, phylogenetic trees were constructed by using the neighbor-joining (NJ), maximum-likelihood (ML), and maximum-parsimony (MP) methods based on the most similar 16S rRNA gene sequences of strains from genus *Sphingobacterium* and representative species from all other 13 genera of the family *Sphingobacteriaceae*. The maximum-likelihood phylogenetic tree analysis indicated that WQ 2009^T^ represented a member of the family *Sphingobacteriaceae*, forming a separate clade within the family *Sphingobacteriaceae* (Fig. 4). Similar topologies were also confirmed in the neighbor-joining tree (Fig. S2) and the maximum-parsimony tree (Fig. S3), suggesting that WQ 2009^T^ should be classified as a new genus of the family *Sphingobacteriaceae*.

**Table 3:** Pairwise comparison of strain WQ 2009^T^ to its closely related strains.

Strain comparison	16S rRNA gene identity (%)	ANI (%)	AAI (%)	dDDH (%)
1/2	94.5	71.3	64.7	13.6
1/3	94.3	70.3	64.9	13.3
1/4	94.3	71.4	65.9	13.8

1, WQ 2009^T^; 2, *S. kitahiroshimense* 10C^T^; 3, *S. pakistanense* NCCP-246^T^; 4, *S. faecium* DSM 11690^T^. ‘*S.*’ stands for ‘*Sphingobacterium*’.

The placement of strain WQ 2009^T^ into a new genus was also supported by the genomic data. The estimated dDDH values between this isolate and *S. kitahiroshimense*, *S. pakistanense* and *S. faecium* were 13.6%, 13.3% and 13.8%, respectively (Table 3), which were far below the commonly used 70% threshold for microbial taxonomy^67^. The calculated ANI values between this isolate and the type strains of its closest taxa were below 71.4%, which was also lower than the threshold (< 74.8%) for genus delineation (Table 3)^69^. Moreover, the AAI values between this isolate and *S. kitahiroshimense*, *S. pakistanense* and *S. faecium* were 64.7%, 64.9% and 65.9%, respectively, which was below or slightly higher than the threshold proposed for a new genus^68^. To confirm the phylogenetic relationship of strain WQ 2009^T^, a maximum-likelihood (ML) phylogenomic tree was constructed on the basis of 670 orthologous genes. WQ 2009^T^ was clearly separated from other genera and formed a distinct branch with a high average branch support of 100% (Fig. S4). Thus, according to the phylogenetic and genomic analysis strain WQ 2009^T^ deserves a representative of a new genus in the family *Sphingobacteriaceae*.

The genome of strain WQ 2009^T^ was 3,144,471 bp with 34 contigs and encoded 2731 genes and 76 tRNAs. The genome size of WQ 2009^T^ was much smaller than that of closely related strains of genus *Sphingobacterium* (5.1–6.7 M), but similar to those of genus *Albibacterium* (3.1 M), *Daejeonella* (3.4 M), and *Solitalea* (3.3 M). The G+C content of the genomic DNA was 39.4%. After comparing and annotating the amino acid sequences of these predicted genes with GO, KEGG, COG, NR, Pfam and Swiss-Prot functional databases, it was shown that the number of coding genes was 1854, 2351, 1859, 2444, 1854 and 822, respectively. Totally 279 secreted proteins were predicted by SignalP and TMHMM. When annotated with the Transporter Classification Database, 117 membrane transport proteins were predicted. Six genomic islands were found in the genome of this species, and no prophages or clustered regularly interspaced short palindromic repeat sequences were found. The genome of WQ 2009^T^ also contained a terpene biosynthetic gene cluster when analysed with antiSMASH. Six genes (GM000637, GM000751, GM001257, GM001350, GM001927 and GM002124) were annotated as potential antibiotic resistant genes.

*Bacteroides* generally have an abundance of CAZymes, which play a pivotal role in the nutrient-microbiota-host interaction^70,71^. There were 166 potential CAZymes (Fig. S5) in WQ 2009^T^ when analysed by using the dbCAN server. The signal peptide prediction revealed that 59 of the 166 CAZymes contained signal peptides. These enzymes might be secreted out of the cell or targeted to specific locations in the cell to perform their functions. The 166 CAZymes were divided into five classes: 37 glycosyl transferases (GTs), 80 glycoside hydrolases (GHs), 14 carbohydrate esterases (CEs), 30 carbohydrate binding modules (CBMs) and 5 auxiliary activities (AAs). No polysaccharide lyase (PLs) was found. GHs were the most abundant CAZymes found in WQ 2009^T^ with 80 genes distributed into 37 different families. The 37 families of GHs comprise a number of enzymes with known activities. These enzymes include α-amylase (EC 3.2.1.1), cellulase (EC 3.3.1.4), lichenase (EC 3.2.1.73), cellobiohydroase (EC 3.2.1.91), xyloglucanendohydrolase (EC 3.2.1.151), α-mannosidase (EC 3.2.1.24), α-fucosidase (EC 3.2.1.51), α-L-rhamnosidase (EC 3.2.1.40), et cetra. The GHs play a key role in carbohydrate metabolism, which hydrolyze complex carbohydrates such as starch, hemicellulose, and cellulose^72^. This was in accordance with the diet of Yunnan snub-nosed monkeys, which mainly live on the lichen plant *U. longissima*^10,14^. CAZymes from the closely related strains and the type strains of all genus from the family *Sphingobacteriaceae* were also analysed. All the six classes of CAZymes (GH, GT, CBM, CE, AA, and PL) were found in the genomes of these strains except for strain WQ 2009^T^, *Albibacterium bauzanese* BZ42^T^, and *Solitalea koreensis* DSM 21342^T^ (Table S8). No PL was found in any of the three strains with much smaller genomes. These results indicated that strains of family *Sphingobacteriaceae* of phyla *Bacteroides* were excellent resources for the discovery of new or highly active CAZymes.

## Conclusion

In this study, the microbial composition and CAZyme profiles of the gut microbiota of Yunnan snub-nosed monkeys were studied by culture-dependent and metagenomic sequencing analyses, and a new genus of the *Sphingobacteriaceae* family from the pure cultures was characterized.

Strain WQ 2009^T^, which was isolated from faeces of the highly endangered Yunnan snub-nosed monkeys (*R. bieti*) endemic to China, was characterized through polyphasic and whole genome analyses. Based on the results, the isolate should represent a novel species of a new genus in family *Sphingobacteriaceae*, for which the name *Rhinopithecimicrobium faecis* gen. nov., sp. nov. is proposed. The descriptions of the *Rhinopithecimicrobium faecis* gen. nov., sp. nov. are given in Table 4.

**Table 4:** Description of the *Rhinopithecimicrobium* gen. nov. and *Rhinopithecimicrobium faecis* sp. nov.

Genus name	*Rhinopithecimicrobium*	–
Species name	–	*Rhinopithecimicrobium faecis*
Genus status	gen.nov	–
Genus etymology	Rhi.no.pi.the.ci.mi.cro´bi.um. N.L. masc. n. *Rhinopithecus*, a monkey genus; N.L. neut. n. *microbium*, a microbe; N.L. neut. n. *Rhinopithecimicrobium*, a microbe isolated from the monkey genus *Rhinopithecus*	–
Type species of the genus	*Rhinopithecimicrobium faecis*	–
Specific epithet	–	*faecis*
Species status	–	sp.nov
Species etymology	–	fae’cis L. gen. fem. n. *faecis*, referring to faecal origin from where the type strain was isolated
Description of the new taxon and diagnostic traits	Cells are aerobic, nonmotile, Gram-stain-negative, and oval-shaped. The major fatty acids are C_15:0_ iso, anteiso-C_15:0_ and Summed Feature 3 (C_16:1_ *ω*7*c*/C_16:1_ *ω*6*c*). The major polar lipid is phosphatidylethanolamine (PE). The predominant menaquinone is MK-7. The DNA G+C content is 39.4%. The genus is a member of the phylum *Bacteroidetes*, class *Sphingobacteriia*, order *Sphingobacteriales*, family *Sphingobacteriaceae*. The type species is *Rhinopitheci faecis*	Cells are aerobic, Gram-stain-negative, nonmotile, and oval-shaped (0.7 ± 0.1 × 1.1–1.4 μm). Oxidase and catalase-positive, urease-negative, H_2_S production-negative. Do not produce indole from triptophane. Nitrate is reduced to nitrogen instead of nitrite. Can assimilate glucose, arabinose, glucosamine and maltose. Growth occurs at 0–35 °C (optimum 20–30 °C), pH 7.0–8.0 (optimum pH 7.0), NaCl 0–2% (optimum 0–1.5%, w/v). Capable of hydrolyzing esculine, *p*NPG, Tween 20, Tween 40, Tween 60 and Tween 80, pectin, starch and cellulose, cannot hydrolyze gelatin. Except for lipase and *β*-glucuronidase, the cells are positive for alkaline phosphatase, esterase (C_4_), esterase lipase (C_8_), leucine arylamidase, valine arylamidase, cystine arylamidase, trypsin, *α*-chymotrypsin, acid phosphstase, naphthol-AS-BI-phosphohydrolase,* α*-galactosidase,* β*-galactosidase, *α*-glucosidase, *β*-glucosidase, N-acetyl-*β*-glucosaminidase, *α*-mannosidase, and *α*-fucosidase. Carbon sources include maltose, cellobiose, gentiobiose, turanose, lactose, melibiose, salicin, glucosamine, mannosamine, galactosamine, glucose, mannose, fructose, galactose, fucose, rhamnose, glycerol, glucose-6-PO_4_, frucose-6-PO_4_, glycyl-l-proline, alanine, serine, galactonic acid lactone, glucuronic acid, lactic acid methyl ester, α-keto-glutaric acid, acetoacetic acid and acetic acid. The type strain is sensitive to ampicillin, cefoperazone, chloramphenicol, ciprofloxacin, clindamycin, erythromycin, gentamicin, kanamycin, norfloxacin, penicillin G, piperacillin and polymyxin B, but resistant to nalidixic acid, aztreonam, amikacin and vancomycin. The major fatty acids are C_15:0_ iso, anteiso-C_15:0_ and Summed Feature 3 (C_16:1_ *ω*7*c*/C_16:1_* ω*6*c*). The predominant polar lipid is phosphatidylethanolamine (PE). The predominant menaquinone is MK-7
Country of origin	–	China
Region of origin	–	Yunnan Snub-nosed Monkey National Park, Yunnan
Date of isolation	–	15/01/2020
Source of isolation	–	Faeces of *Rhinopithecus bieti*
Date of Sampling	–	15/10/2019
Latitude	–	27°39′N
Longitude	–	99°21′E
16S rRNA gene accession number	–	MZ413266
Genome accession number	–	JAGKSB000000000.1
Genome status	–	Draft
Genome size	–	3.1 Mb
GC %	–	39.4
Number of strains in study	–	1
Information related to the Nagoya protocol	–	Not applicable
Designation of the type strain	–	WQ 2009^T^
Strain collection numbers	–	CCTCC AB 2021153 = KCTC 82941

### Supplementary Information

Supplementary Information.

## Data Availability

The datasets used and/or analysed during the current study available from the corresponding author on reasonable request.

## Abbreviations

ANI: Average nucleotide identity
LA: Luria–Bertani agar
TSA: Trypticase soy agar
TSB: Trypticase soy broth
TLC: Thin layer chromatography
KCTC: Korean collection for type cultures
CCTCC: China center for type culture collection
TEM: Transmission electron microscope
PCR: Polymerase chain reaction
MK-7: Menaquinone 7
