# Supplementary material for: Metagenomic and culture-dependent analysis of Rhinopithecius bieti gut microbiota and characterization of a novel genus of Sphingobacteriaceae
Source: Sci Rep. 2024 Jun 15;14:13819. doi: 10.1038/s41598-024-64727-9 (PMC11180105; doi:10.1038/s41598-024-64727-9)
Supplement: Supplementary file 1 — Supplementary Information. [file 41598_2024_64727_MOESM1_ESM.docx]

**Supplementary materials**

**Metagenomic and culture-dependent analysis of *Rhinopithecius bieti* gut microbiota and characterization of a novel genus of *Sphingobacteriaceae***

Qiong Wang^1^, Peng-Chao Zhan, Xiu-Lin Han^*^, Tao Lu^*^

**Affiliation**

Yunnan Institute of Microbiology, Key Laboratory for Southwest Microbial Diversity of the Ministry of Education, School of Life Sciences, Yunnan University, Kunming, Yunnan 650500, PR China

**Corresponding author**

*Tao Lu, [taolu3000@163.com](mailto:taolu3000@163.com); Xiu-Lin Han, xlhan@ynu.edu.cn.

^1^ Present address: Center for Pharmaceutical Sciences, Faculty of Life Science and Technology, Kunming University of Science and Technology, Kunming, Yunnan 650500, PR China

**Fig. S1** The major polar lipids of strain WQ 2009^T^. PE, phosphatidylethanolamine; PL, unknown phospholipids; GPL, unknown glycophospholipid; GL, unknown glycolipid; L, unknown lipid.


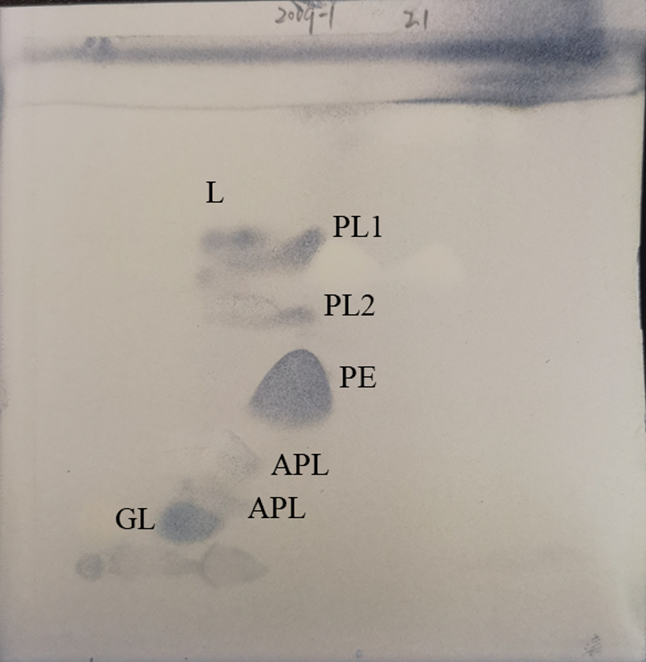


**Fig. S2** The neighbor-joining phylogenetic tree based on the 16S rRNA gene sequence of strain WQ 2009^T^, showing the taxonomic position of the strain and the closely related taxa. Bootstrap values (expressed as percentages of 1000 replications) of above 50 % are shown at branch points. *Filobacterium rodentium* SMR-C^T^ was used as the outgroup. Bar, 0.02 substitutions per nucleotide position.

67

5 *Sphingobacterium* spp.

*Sphingobacterium pakistanense* NCCP-246^T^ (AB610802)

*Sphingobacterium faecium* DSM 11690^T^ (AJ438176)

*Sphingobacterium kitahiroshimense* 10C^T^ (AB361248)

*Sphingobacterium anhuiense* CW186^T^ (EU364817)

**WQ 2009^T^ (MT951305)**

*Albibacterium bauzanense* BZ42^T^ (GQ161990)

*Pseudopedobacter beijingensis* GCS-AE-31^T^ (KC755039)

*Mucilaginibacter agri* R11^T^ (MN080907)

*Mucilaginibacter paludis* TPT56^T^ (AM490402)

*Pedobacter daechungensis* Dae 13^T^ (AB267722)

*Pedobacter bambusae* THG-G118^T^ (KF150694)

*Daejeonella oryzae* DSM 19973^T^ (AUHA01000011)

*Arcticibacter svalbardensis* MN12-7^T^ (JQ396621)

*Pararcticibacter amylolyticus* FJ4-8^T^ (MG719992)

*Parapedobacter soli* DCY14T (EF151805)

*Olivibacter composti* CC-KYC063^T^ (KU358715)

*Nubsella zeaxanthinifaciens* TDMA-5^T^ (AB264126)

*Pelobium manganitolerans* YS-25^T^ (KX014810)

*Solitalea canadensis* DSM 3403^T^ (AB078046)

*Filobacterium rodentium* SMR-C^T^ (LC055729)

92

100

100

99

75

50

91

91

59

60

54

92

0.020

**Fig. S3** The phylogenetic tree based on 16S rRNA gene sequences using the maximum-parsimony method. Bootstrap values (expressed as percentages of 1000 replications) of above 50 % are shown at branch points. *Filobacterium rodentium* SMR-C^T^ was used as the out-group. That is, fewer than 2% alignment gaps, missing data, and ambiguous bases were allowed at any position.

4 *Sphingobacterium* spp.

*Sphingobacterium ginsenosidimutans* THG 07^T^ (GU138378)

*Sphingobacterium pakistanense* NCCP-246^T^ (AB610802)

*Sphingobacterium faecium* DSM 11690^T^ (AJ438176)

*Sphingobacterium kitahiroshimense* 10C^T^ (AB361248)

*Sphingobacterium anhuiense* CW186^T^ (EU364817)

**WQ 2009^T^ (MT951305)**

*Albibacterium bauzanense* BZ42^T^ (GQ161990)

*Pedobacter daechungensis* Dae 13^T^ (AB267722)

*Pedobacter bambusae* THG-G118^T^ (KF150694)

*Daejeonella oryzae* DSM 19973^T^ (AUHA01000011)

*Mucilaginibacter agri* R11^T^ (MN080907)

*Mucilaginibacter paludis* TPT56^T^ (AM490402)

*Pararcticibacter amylolyticus* FJ4-8^T^ (MG719992)

*Nubsella zeaxanthinifaciens* TDMA-5^T^ (AB264126)

*Pseudopedobacter beijingensis* GCS-AE-31^T^ (KC755039)

*Pelobium manganitolerans* YS-25^T^ (KX014810)

*Parapedobacter soli* DCY14^T^ (EF151805)

*Olivibacter composti* CC-KYC063^T^ (KU358715)

*Arcticibacter svalbardensis* MN12-7^T^ (JQ396621)

*Solitalea canadensis* DSM 3403^T^ (AB078046)

*Filobacterium rodentium* SMR-C^T^ (LC055729)

87

92

100

93

60

53

100

76

59

99

78

**Fig. S4** The maximun-likelihood phylogenomic tree of WQ 2009 based on the concatenated conserved blocks of 670 orthologous genes. *Solitalea koreensis* DSM 21342^T^ (GCA_900182575.1) and *Anseongella ginsenosidimutangs* DSM 21100^T^ (GCA_004346135.1) were used as an out-group for phylogenetic analysis. The numbers shown along branches indicate the bootstrap support expressed as percentages.


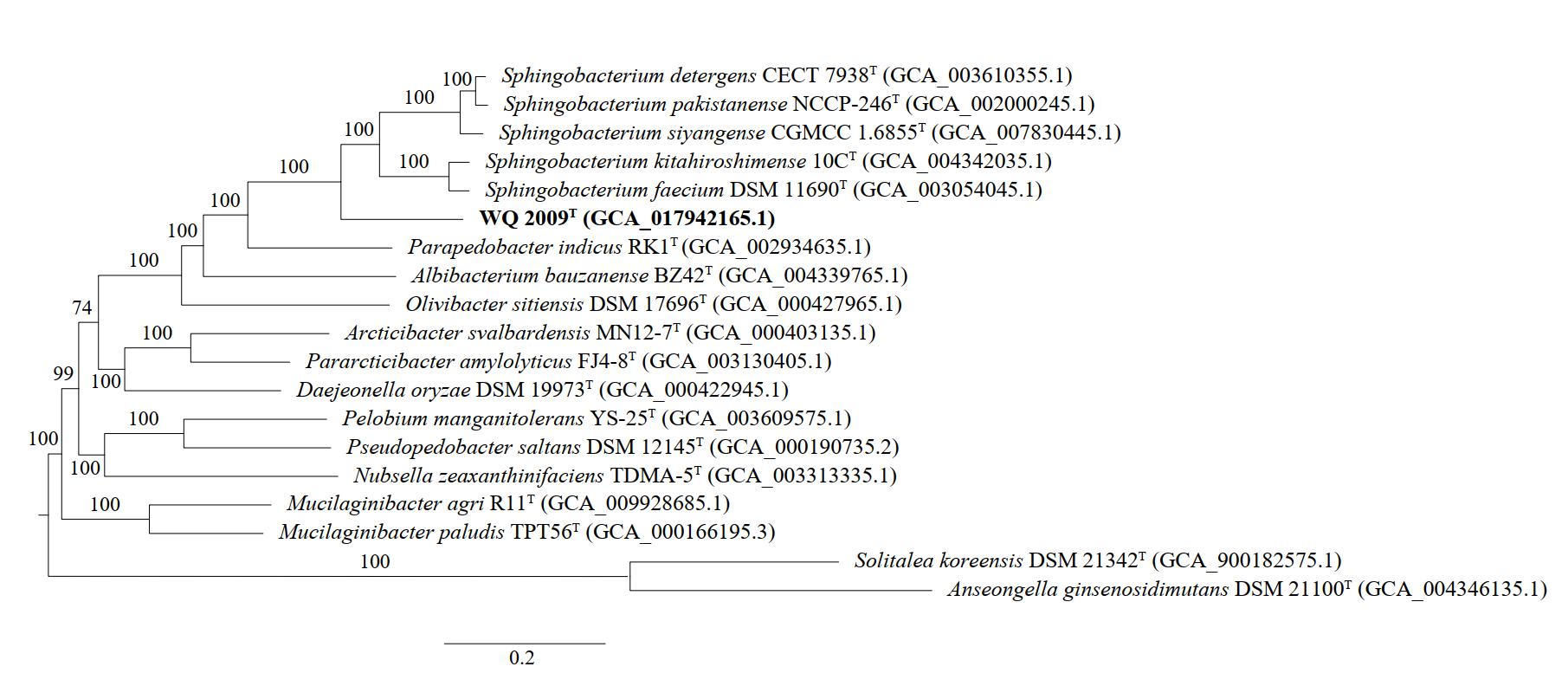


**Fig. S5** Distribution of CAZymes in WQ 2009^T^. From inner to outer rings: CAZyme classes, CAZyme families within each class, and the number of genes belonging to each family.


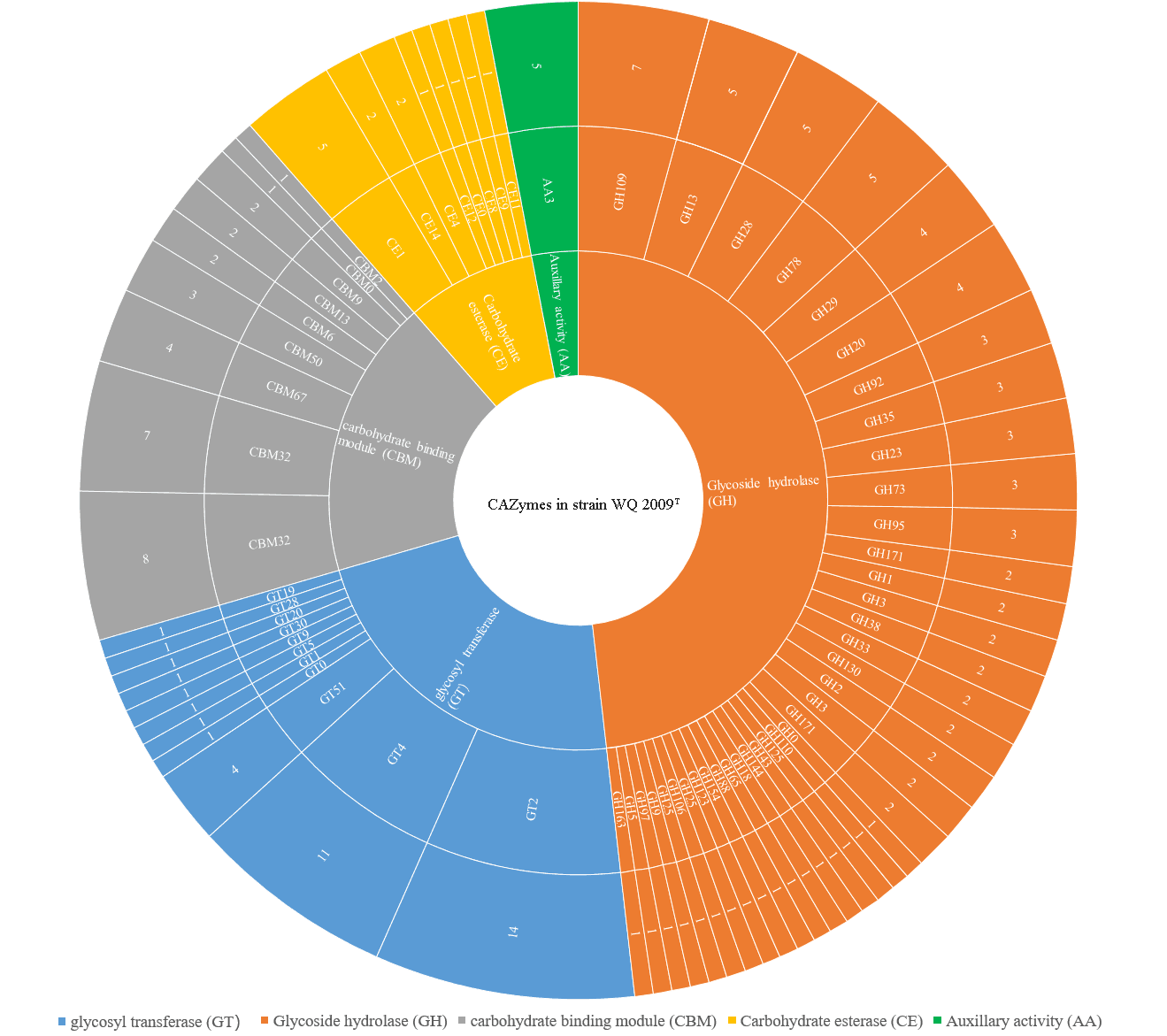


**Table S1 Quality estimation of MAGs**

| genome | completeness (%) | contamination (%) | genome | completeness (%) | contamination (%) | genome | completeness (%) | contamination (%) | genome | completeness (%) | contamination (%) |
| --- | --- | --- | --- | --- | --- | --- | --- | --- | --- | --- | --- |
| bin.80 | 100 | 0 | bin.44 | 93.38 | 0 | bin.79 | 85.26 | 2 | bin.195 | 71.31 | 4.37 |
| bin.175 | 99.43 | 0.02 | bin.169 | 93.22 | 2.98 | bin.45 | 84.54 | 0.34 | bin.48 | 71.02 | 0.67 |
| bin.46 | 98.82 | 0 | bin.14 | 93.07 | 0.34 | bin.103 | 84.52 | 0 | bin.126 | 69.45 | 6.11 |
| bin.84 | 97.81 | 0.75 | bin.4 | 93.06 | 0.19 | bin.65 | 84.5 | 0.48 | bin.50 | 69.43 | 0.38 |
| bin.163 | 97.28 | 0.68 | bin.2 | 92.94 | 1.19 | bin.131 | 84.36 | 3.59 | bin.171 | 67.86 | 4.77 |
| bin.72 | 97.2 | 0.7 | bin.58 | 92.66 | 2.91 | bin.192 | 84.26 | 8.39 | bin.157 | 67.62 | 0 |
| bin.113 | 97.09 | 0.67 | bin.155 | 92.34 | 0 | bin.156 | 83.5 | 2.35 | bin.139 | 65.17 | 0.56 |
| bin.179 | 96.98 | 0.38 | bin.75 | 92.03 | 0.89 | bin.38 | 83.07 | 0.13 | bin.73 | 64.23 | 0.67 |
| bin.123 | 96.87 | 0 | bin.196 | 91.67 | 6.04 | bin.91 | 83 | 0.92 | bin.12 | 63.53 | 2.76 |
| bin.96 | 96.84 | 2.14 | bin.117 | 91.67 | 0.48 | bin.122 | 82.99 | 4.08 | bin.63 | 63.44 | 1.07 |
| bin.151 | 96.7 | 1.65 | bin.118 | 91.18 | 3.75 | bin.25 | 82.84 | 0.81 | bin.183 | 62.24 | 0.7 |
| bin.159 | 96.69 | 1.01 | bin.147 | 91.17 | 0.53 | bin.20 | 82.34 | 1.51 | bin.57 | 61.8 | 0.81 |
| bin.59 | 96.09 | 1.51 | bin.166 | 91.13 | 0.03 | bin.90 | 82.25 | 1.05 | bin.108 | 61.29 | 0 |
| bin.5 | 96.04 | 4.15 | bin.107 | 91.04 | 0 | bin.8 | 80.01 | 0.39 | bin.142 | 60.75 | 0 |
| bin.71 | 96 | 0 | bin.86 | 90.6 | 4.51 | bin.190 | 79.7 | 0 | bin.87 | 59.97 | 3.76 |
| bin.121 | 95.97 | 1.48 | bin.105 | 90.58 | 0.39 | bin.138 | 79.69 | 3.47 | bin.52 | 59.82 | 0.88 |
| bin.115 | 95.71 | 0.89 | bin.64 | 90.2 | 7.18 | bin.10 | 78.6 | 3.21 | bin.85 | 59.79 | 2.03 |
| bin.174 | 95.08 | 0 | bin.94 | 89.81 | 0.06 | bin.188 | 78.49 | 0.5 | bin.148 | 59.69 | 1.34 |
| bin.162 | 94.91 | 0.96 | bin.89 | 88.76 | 1.12 | bin.134 | 78.07 | 1.21 | bin.173 | 58.87 | 0.86 |
| bin.42 | 94.83 | 8.62 | bin.76 | 88.74 | 0.69 | bin.9 | 76.93 | 3.69 | bin.24 | 57.07 | 9.89 |
| bin.130 | 94.81 | 0.95 | bin.7 | 87.96 | 5.62 | bin.129 | 76.02 | 0.1 | bin.34 | 56.9 | 1.72 |
| bin.33 | 94.73 | 1.93 | bin.55 | 87.88 | 2.51 | bin.95 | 75.56 | 8.63 | bin.70 | 56.77 | 4.37 |
| bin.3 | 94.72 | 0 | bin.54 | 87.64 | 1.69 | bin.39 | 75.56 | 0.51 | bin.29 | 56.72 | 0 |
| bin.13 | 94.62 | 2.69 | bin.185 | 87.25 | 1.34 | bin.6 | 74.56 | 1.7 | bin.176 | 56.39 | 2.51 |
| bin.93 | 94.13 | 0.56 | bin.36 | 87.16 | 1.38 | bin.49 | 74.39 | 2.46 | bin.111 | 53.8 | 1.34 |
| bin.69 | 93.84 | 1.05 | bin.124 | 86.42 | 1.38 | bin.125 | 74.28 | 1.14 | bin.53 | 52.63 | 3.45 |
| bin.180 | 93.84 | 0.38 | bin.191 | 86.24 | 0 | bin.119 | 72.73 | 3.45 | bin.77 | 52.28 | 0 |
| bin.78 | 93.55 | 0 | bin.16 | 85.7 | 3.08 | bin.56 | 71.83 | 0.81 | bin.143 | 51.88 | 0 |
| bin.167 | 93.45 | 1.88 | bin.43 | 85.44 | 1.57 | bin.11 | 71.78 | 0 |  |  |  |

**Table S2 Taxonomy of MAGs**

| genome | domain | phylum | class | order | family | genus | species |
| --- | --- | --- | --- | --- | --- | --- | --- |
| bin.124 | Bacteria | Verrucomicrobiota | Kiritimatiellia | RFP12 | UBA1067 | UBA1731 | Unclassified |
| bin.122 | Bacteria | Verrucomicrobiota | Kiritimatiellia | RFP12 | UBA1067 | UBA1067 | Unclassified |
| bin.36 | Bacteria | Verrucomicrobiota | Kiritimatiellia | RFP12 | UBA1067 | RUG572 | Unclassified |
| bin.118 | Bacteria | Verrucomicrobiota | Verrucomicrobiae | Opitutales | CAG-312 | Merdousia | Merdousia sp900760055 |
| bin.163 | Bacteria | Verrucomicrobiota | Verrucomicrobiae | Verrucomicrobiales | Akkermansiaceae | Akkermansia | Unclassified |
| bin.183 | Bacteria | Spirochaetota | Spirochaetia | Treponematales | Treponemataceae | Treponema_D | Unclassified |
| bin.70 | Bacteria | Spirochaetota | Spirochaetia | Treponematales | Treponemataceae | Treponema_D | Unclassified |
| bin.72 | Bacteria | Spirochaetota | Spirochaetia | Treponematales | Treponemataceae | Treponema_D | Unclassified |
| bin.63 | Bacteria | Spirochaetota | Spirochaetia | Sphaerochaetales | Sphaerochaetaceae | Ornithospirochaeta | Unclassified |
| bin.71 | Bacteria | Spirochaetota | Brachyspirae | Brachyspirales | Brachyspiraceae | Brachyspira | Unclassified |
| bin.151 | Bacteria | Pseudomonadota | Alphaproteobacteria | RF32 | CAG-239 | CAG-267 | Unclassified |
| bin.125 | Bacteria | Pseudomonadota | Alphaproteobacteria | RF32 | CAG-239 | Unclassfied | Unclassified |
| bin.80 | Archaea | Methanobacteriota | Methanobacteria | Methanobacteriales | Methanobacteriaceae | Methanobrevibacter_A | Methanobrevibacter_A smithii |
| bin.129 | Bacteria | Fibrobacterota | Fibrobacteria | Fibrobacterales | Fibrobacteraceae | Hallerella | Unclassified |
| bin.46 | Bacteria | Desulfobacterota | Desulfovibrionia | Desulfovibrionales | Desulfovibrionaceae | Desulfovibrio | Desulfovibrio porci |
| bin.147 | Bacteria | Campylobacterota | Campylobacteria | Campylobacterales | Helicobacteraceae | NHYM01 | Unclassified |
| bin.44 | Bacteria | Campylobacterota | Campylobacteria | Campylobacterales | Campylobacteraceae | Campylobacter_D | Unclassified |
| bin.12 | Bacteria | Bacteroidota | Bacteroidia | Bacteroidales | Muribaculaceae | UBA7173 | Unclassified |
| bin.69 | Bacteria | Bacteroidota | Bacteroidia | Bacteroidales | Bacteroidaceae | UBA1786 | Unclassified |
| bin.5 | Bacteria | Bacteroidota | Bacteroidia | Bacteroidales | Muribaculaceae | RGIG4576 | Unclassified |
| bin.77 | Bacteria | Bacteroidota | Bacteroidia | Bacteroidales | Bacteroidaceae | Prevotella | Unclassified |
| bin.4 | Bacteria | Bacteroidota | Bacteroidia | Bacteroidales | Bacteroidaceae | Phocaeicola | Unclassified |
| bin.13 | Bacteria | Bacteroidota | Bacteroidia | Bacteroidales | P3 | Phil12 | Unclassified |
| bin.42 | Bacteria | Bacteroidota | Bacteroidia | Bacteroidales | P3 | Phil12 | Unclassified |
| bin.142 | Bacteria | Bacteroidota | Bacteroidia | Bacteroidales | Muribaculaceae | Paramuribaculum | Unclassified |
| bin.39 | Bacteria | Bacteroidota | Bacteroidia | Bacteroidales | Tannerellaceae | Parabacteroides | Parabacteroides distasonis |
| bin.143 | Bacteria | Bacteroidota | Bacteroidia | Bacteroidales | Muribaculaceae | JAJPXC01 | Unclassified |
| bin.50 | Bacteria | Bacteroidota | Bacteroidia | Bacteroidales | Muribaculaceae | JAGBWK01 | Unclassified |
| bin.180 | Bacteria | Bacteroidota | Bacteroidia | Bacteroidales | Muribaculaceae | Duncaniella | Unclassified |
| bin.117 | Bacteria | Bacteroidota | Bacteroidia | Bacteroidales | UBA932 | Cryptobacteroides | Unclassified |
| bin.130 | Bacteria | Bacteroidota | Bacteroidia | Bacteroidales | UBA932 | Cryptobacteroides | Unclassified |
| bin.2 | Bacteria | Bacteroidota | Bacteroidia | Bacteroidales | UBA932 | Cryptobacteroides | Unclassified |
| bin.65 | Bacteria | Bacteroidota | Bacteroidia | Bacteroidales | UBA932 | Cryptobacteroides | Unclassified |
| bin.179 | Bacteria | Bacteroidota | Bacteroidia | Bacteroidales | Muribaculaceae | CAG-873 | Unclassified |
| bin.188 | Bacteria | Bacteroidota | Bacteroidia | Bacteroidales | Muribaculaceae | CAG-873 | Unclassified |
| bin.59 | Bacteria | Bacteroidota | Bacteroidia | Bacteroidales | Muribaculaceae | CAG-485 | Unclassified |
| bin.84 | Bacteria | Bacteroidota | Bacteroidia | Bacteroidales | Bacteroidaceae | Bacteroides | Bacteroides intestinalis |
| bin.79 | Bacteria | Bacteroidota | Bacteroidia | Bacteroidales | Rikenellaceae | Alistipes | Alistipes sp900548155 |
| bin.52 | Bacteria | Bacillota_C | Negativicutes | Acidaminococcales | Acidaminococcaceae | Phascolarctobacterium_A | Unclassified |
| bin.86 | Bacteria | Bacillota_C | Negativicutes | Acidaminococcales | Acidaminococcaceae | Phascolarctobacterium_A | Unclassified |
| bin.55 | Bacteria | Bacillota_A | Clostridia | Lachnospirales | Lachnospiraceae | VSOB01 | Unclassified |
| bin.195 | Bacteria | Bacillota_A | Clostridia | Oscillospirales | Oscillospiraceae | Vescimonas | Unclassified |
| bin.20 | Bacteria | Bacillota_A | Clostridia | Oscillospirales | Oscillospiraceae | Vescimonas | Unclassified |
| bin.196 | Bacteria | Bacillota_A | Clostridia | Christensenellales | CAG-74 | Ventricola | Unclassified |
| bin.11 | Bacteria | Bacillota_A | Clostridia | Christensenellales | UBA1242 | UMGS1908 | Unclassified |
| bin.111 | Bacteria | Bacillota_A | Clostridia | Oscillospirales | CAG-272 | UMGS1696 | Unclassified |
| bin.134 | Bacteria | Bacillota_A | Clostridia | Christensenellales | UBA1242 | UMGS1470 | Unclassified |
| bin.173 | Bacteria | Bacillota_A | Clostridia | Oscillospirales | Oscillospiraceae | UBA738 | UBA738 sp004557735 |
| bin.75 | Bacteria | Bacillota_A | Clostridia | Oscillospirales | Acutalibacteraceae | UBA737 | Unclassified |
| bin.176 | Bacteria | Bacillota_A | Clostridia | Lachnospirales | Lachnospiraceae | UBA7050 | Unclassified |
| bin.156 | Bacteria | Bacillota_A | Clostridia | Oscillospirales | Acutalibacteraceae | UBA6857 | Unclassified |
| bin.95 | Bacteria | Bacillota_A | Clostridia | Christensenellales | Borkfalkiaceae | UBA4636 | Unclassified |
| bin.175 | Bacteria | Bacillota_A | Clostridia | Lachnospirales | Lachnospiraceae | UBA3766 | UBA3766 sp015057025 |
| bin.85 | Bacteria | Bacillota_A | Clostridia | Oscillospirales | CAG-382 | UBA1752 | Unclassified |
| bin.185 | Bacteria | Bacillota_A | Clostridia | Oscillospirales | CAJFTT01 | Tabaqchalia | Unclassified |
| bin.58 | Bacteria | Bacillota_A | Clostridia | Lachnospirales | Lachnospiraceae | SIG307 | Unclassified |
| bin.108 | Bacteria | Bacillota_A | Clostridia | Christensenellales | CAG-74 | SFTH01 | Unclassified |
| bin.53 | Bacteria | Bacillota_A | Clostridia | Christensenellales | CAG-74 | SFTH01 | Unclassified |
| bin.64 | Bacteria | Bacillota_A | Clostridia | Christensenellales | CAG-74 | SFTH01 | Unclassified |
| bin.87 | Bacteria | Bacillota_A | Clostridia | Christensenellales | CAG-74 | SFTH01 | Unclassified |
| bin.10 | Bacteria | Bacillota_A | Clostridia | Christensenellales | CAG-138 | SFEL01 | SFEL01 sp004557245 |
| bin.90 | Bacteria | Bacillota_A | Clostridia | Christensenellales | CAG-138 | SFEL01 | Unclassified |
| bin.155 | Bacteria | Bacillota_A | Clostridia | Christensenellales | CAG-314 | SFEB01 | SFEB01 sp004558105 |
| bin.121 | Bacteria | Bacillota_A | Clostridia | Christensenellales | Borkfalkiaceae | Scatosoma | Unclassified |
| bin.78 | Bacteria | Bacillota_A | Clostridia | Christensenellales | Borkfalkiaceae | Scatosoma | Unclassified |
| bin.113 | Bacteria | Bacillota_A | Clostridia | Oscillospirales | Ruminococcaceae | Ruminiclostridium_E | Unclassified |
| bin.167 | Bacteria | Bacillota_A | Clostridia | Christensenellales | CAG-74 | RUG563 | Unclassified |
| bin.103 | Bacteria | Bacillota_A | Clostridia | Christensenellales | CAG-138 | RUG472 | Unclassified |
| bin.162 | Bacteria | Bacillota_A | Clostridia | Lachnospirales | Lachnospiraceae | RGIG8767 | Unclassified |
| bin.148 | Bacteria | Bacillota_A | Clostridia | Oscillospirales | Acutalibacteraceae | RGIG7067 | Unclassified |
| bin.157 | Bacteria | Bacillota_A | Clostridia | Oscillospirales | CAG-272 | RGIG4608 | Unclassified |
| bin.43 | Bacteria | Bacillota_A | Clostridia | Christensenellales | CAG-917 | RGIG4097 | Unclassified |
| bin.57 | Bacteria | Bacillota_A | Clostridia | Christensenellales | CAG-138 | PeH17 | PeH17 sp004556165 |
| bin.171 | Bacteria | Bacillota_A | Clostridia | Lachnospirales | Lachnospiraceae | Pararoseburia | Unclassified |
| bin.89 | Bacteria | Bacillota_A | Clostridia | Christensenellales | UBA1242 | Onthoplasma | Unclassified |
| bin.38 | Bacteria | Bacillota_A | Clostridia | Oscillospirales | Oscillospiraceae | Limivicinus | Unclassified |
| bin.45 | Bacteria | Bacillota_A | Clostridia | Oscillospirales | Oscillospiraceae | Limivicinus | Unclassified |
| bin.192 | Bacteria | Bacillota_A | Clostridia | Christensenellales | CAG-74 | Limiplasma | Unclassified |
| bin.49 | Bacteria | Bacillota_A | Clostridia | Christensenellales | CAG-74 | Limiplasma | Unclassified |
| bin.3 | Bacteria | Bacillota_A | Clostridia | Peptostreptococcales | Anaerovoracaceae | Lentihominibacter | Unclassified |
| bin.29 | Bacteria | Bacillota_A | Clostridia | Oscillospirales | CAG-272 | HGM12650 | Unclassified |
| bin.119 | Bacteria | Bacillota_A | Clostridia | Christensenellales | Borkfalkiaceae | HGM11416 | Unclassified |
| bin.166 | Bacteria | Bacillota_A | Clostridia | Christensenellales | CAG-314 | Fimimonas | Unclassified |
| bin.91 | Bacteria | Bacillota_A | Clostridia | Christensenellales | CAG-314 | Fimimonas | Unclassified |
| bin.105 | Bacteria | Bacillota_A | Clostridia | Oscillospirales | Oscillospiraceae | Faecousia | Unclassified |
| bin.16 | Bacteria | Bacillota_A | Clostridia | Oscillospirales | Oscillospiraceae | Faecousia | Unclassified |
| bin.34 | Bacteria | Bacillota_A | Clostridia | Oscillospirales | Oscillospiraceae | Faecousia | Unclassified |
| bin.94 | Bacteria | Bacillota_A | Clostridia | Oscillospirales | Oscillospiraceae | Faecousia | Unclassified |
| bin.96 | Bacteria | Bacillota_A | Clostridia | Lachnospirales | Lachnospiraceae | Faecalimonas | Unclassified |
| bin.76 | Bacteria | Bacillota_A | Clostridia | Oscillospirales | Oscillospiraceae | F23-B02 | Unclassified |
| bin.174 | Bacteria | Bacillota_A | Clostridia | Oscillospirales | Acutalibacteraceae | DTU089 | Unclassified |
| bin.123 | Bacteria | Bacillota_A | Clostridia | Oscillospirales | CAG-272 | DTU064 | Unclassified |
| bin.159 | Bacteria | Bacillota_A | Clostridia | Oscillospirales | Acutalibacteraceae | Caproiciproducens | Unclassified |
| bin.131 | Bacteria | Bacillota_A | Clostridia | Lachnospirales | Lachnospiraceae | CALWPC01 | Unclassified |
| bin.190 | Bacteria | Bacillota_A | Clostridia | Christensenellales | UBA1242 | CAKVLS01 | Unclassified |
| bin.191 | Bacteria | Bacillota_A | Clostridia | Oscillospirales | Oscillospiraceae | CAJOIG01 | Unclassified |
| bin.73 | Bacteria | Bacillota_A | Clostridia | Oscillospirales | Oscillospiraceae | CAJOIG01 | Unclassified |
| bin.33 | Bacteria | Bacillota_A | Clostridia | Lachnospirales | Lachnospiraceae | CAG-95 | CAG-95 sp017434885 |
| bin.8 | Bacteria | Bacillota_A | Clostridia | Christensenellales | CAG-917 | CAG-475 | Unclassified |
| bin.138 | Bacteria | Bacillota_A | Clostridia | Oscillospirales | CAG-272 | CAG-448 | Unclassified |
| bin.25 | Bacteria | Bacillota_A | Clostridia | Oscillospirales | CAG-272 | CAG-448 | Unclassified |
| bin.14 | Bacteria | Bacillota_A | Clostridia | Oscillospirales | CAG-272 | CAG-390 | CAG-390 sp003523225 |
| bin.6 | Bacteria | Bacillota_A | Clostridia | Oscillospirales | CAG-272 | CAG-390 | Unclassified |
| bin.115 | Bacteria | Bacillota_A | Clostridia | Oscillospirales | Acutalibacteraceae | CAG-177 | Unclassified |
| bin.93 | Bacteria | Bacillota_A | Clostridia | Oscillospirales | Acutalibacteraceae | CAG-177 | Unclassified |
| bin.24 | Bacteria | Bacillota_A | Clostridia | Christensenellales | CAG-138 | CAG-1024 | CAG-1024 sp004554165 |
| bin.56 | Bacteria | Bacillota_A | Clostridia | Christensenellales | UBA1242 | Caccovivens | Unclassified |
| bin.48 | Bacteria | Bacillota_A | Clostridia | Oscillospirales | CAG-272 | Unclassified | Unclassified |
| bin.9 | Bacteria | Bacillota_A | Clostridia | Oscillospirales | CAG-272 | Unclassified | Unclassified |
| bin.169 | Bacteria | Bacillota | Bacilli | Bacillales_K | Sporolactobacillaceae | Sporolactobacillus | Unclassified |
| bin.7 | Bacteria | Bacillota | Bacilli | RF39 | UBA660 | RUG591 | Unclassified |
| bin.126 | Bacteria | Bacillota | Bacilli | Lactobacillales | Enterococcaceae | Enterococcus_B | Enterococcus_B durans |
| bin.54 | Bacteria | Bacillota | Bacilli | RF39 | UBA660 | CAG-914 | CAG-914 sp000437895 |
| bin.139 | Bacteria | Bacillota | Bacilli | RF39 | UBA660 | CAG-533 | Unclassified |
| bin.107 | Bacteria | Bacillota | Bacilli | Erysipelotrichales | Erysipelotrichaceae | Amedibacterium | Unclassified |

**Table S3** Abundance of MAGs in the faecal samples of *R. bieti* (genus with abundance greater than 1% were displayed).

| Genus | Relative abundance | Percent (%) | Phylum | Relative abundance | Percent (%) |
| --- | --- | --- | --- | --- | --- |
| *Cryptobacteroides* | 171073.14 | 17.11 | *Bacillota_A* | 451720.23 | 45.17 |
| *CAG-914* | 60473.24 | 6.05 | *Bacteroidota* | 298176.48 | 29.82 |
| *CAJOIG01* | 38065.99 | 3.81 | *Bacillota* | 80267.63 | 8.03 |
| *DTU089* | 36587.77 | 3.66 | *Verrucomicrobiota* | 46836.44 | 4.68 |
| *Treponema_D* | 29738.18 | 2.97 | *Spirochaetota* | 45720.75 | 4.57 |
| *Faecousia* | 28951.19 | 2.90 | *Campylobacterota* | 26889.03 | 2.69 |
| *JAGBWK01* | 22472.56 | 2.25 | *Desulfobacterota* | 17830.85 | 1.78 |
| *Onthoplasma* | 22021.72 | 2.20 | *Methanobacteriota* | 16674.49 | 1.67 |
| *RGIG4608* | 20667.47 | 2.07 | *Pseudomonadota* | 9191.33 | 0.92 |
| *UBA3766* | 19998.18 | 2.00 | *Bacillota_C* | 4703.77 | 0.47 |
| *Phocaeicola* | 19190.77 | 1.92 | *Fibrobacterota* | 1989.06 | 0.20 |
| *Akkermansia* | 18118.62 | 1.81 |  |  |  |
| *Desulfovibrio* | 17830.85 | 1.78 |  |  |  |
| *CAG-873* | 16915.66 | 1.69 |  |  |  |
| *CAKVLS01* | 16785.58 | 1.68 |  |  |  |
| *Methanobrevibacter_A* | 16674.49 | 1.67 |  |  |  |
| *RUG472* | 15561.04 | 1.56 |  |  |  |
| *PeH17* | 15318.29 | 1.53 |  |  |  |
| *Prevotella* | 15263.03 | 1.53 |  |  |  |
| *Brachyspira* | 14209.99 | 1.42 |  |  |  |
| *Campylobacter_D* | 14119.40 | 1.41 |  |  |  |
| *Vescimonas* | 13368.28 | 1.34 |  |  |  |
| *CAG-177* | 12971.21 | 1.30 |  |  |  |
| *NHYM01* | 12769.63 | 1.28 |  |  |  |
| *Limiplasma* | 12531.07 | 1.25 |  |  |  |
| *RUG572* | 11534.76 | 1.15 |  |  |  |
| *UBA1731* | 11515.15 | 1.15 |  |  |  |
| *SFTH01* | 11112.51 | 1.11 |  |  |  |
| *Phil12* | 10742.64 | 1.07 |  |  |  |
| *Limivicinus* | 10577.47 | 1.06 |  |  |  |
| *Fimimonas* | 10570.02 | 1.06 |  |  |  |

**Table S4** Summary of CAZyme distribution in the metagenome of *R. bieti*.

| 112 GH  Family  (10,167) | Family | No. | Family | No. | Family | No. | Family | No. | Family | No. | Family | No. | Family | No. | Family | No. | Family | No. |
| --- | --- | --- | --- | --- | --- | --- | --- | --- | --- | --- | --- | --- | --- | --- | --- | --- | --- | --- |
|  | GH2 | 1026 | GH20 | 218 | GH105 | 130 | GH109 | 64 | GH53 | 41 | GH8 | 19 | GH104 | 11 | GH59 | 5 | GH98 | 2 |
|  | GH3 | 708 | GH92 | 213 | GH95 | 130 | GH26 | 64 | GH89 | 39 | GH79 | 17 | GH148 | 11 | GH102 | 4 | GH114 | 1 |
|  | GH43 | 653 | GH29 | 188 | GH97 | 125 | GH42 | 63 | GH76 | 33 | GH140 | 16 | GH85 | 10 | GH147 | 4 | GH129 | 1 |
|  | GH13 | 465 | GH55 | 174 | GH16 | 119 | GH9 | 62 | GH146 | 32 | GH142 | 16 | GH91 | 10 | GH17 | 4 | GH44 | 1 |
|  | GH28 | 384 | GH18 | 173 | GH10 | 117 | GH106 | 61 | GH63 | 31 | GH19 | 16 | GH11 | 8 | GH45 | 4 | GH6 | 1 |
|  | GH78 | 334 | GH72 | 170 | GH4 | 98 | GH112 | 52 | GH110 | 30 | GH117 | 14 | GH145 | 8 | GH139 | 3 | GH64 | 1 |
|  | GH77 | 316 | GH38 | 161 | GH65 | 87 | GH39 | 52 | GH84 | 27 | GH137 | 14 | GH103 | 7 | GH149 | 3 | GH81 | 1 |
|  | GH23 | 267 | GH5 | 160 | GH130 | 86 | GH125 | 50 | GH67 | 25 | GH136 | 13 | GH108 | 7 | GH37 | 3 | GH86 | 1 |
|  | GH94 | 254 | GH33 | 149 | GH27 | 81 | GH24 | 49 | GH144 | 23 | GH141 | 13 | GH138 | 7 | GH71 | 3 |  |  |
|  | GH31 | 250 | GH51 | 148 | GH127 | 70 | GH49 | 47 | GH47 | 23 | GH74 | 13 | GH113 | 6 | GH87 | 3 |  |  |
|  | GH73 | 240 | GH1 | 142 | GH30 | 69 | GH88 | 47 | GH120 | 22 | GH101 | 12 | GH128 | 6 | GH12 | 2 |  |  |
|  | GH36 | 229 | GH35 | 141 | GH133 | 68 | GH115 | 44 | GH50 | 22 | GH143 | 12 | GH15 | 6 | GH121 | 2 |  |  |
|  | GH25 | 221 | GH32 | 131 | GH57 | 65 | GH123 | 42 | GH116 | 21 | GH151 | 12 | GH66 | 6 | GH93 | 2 |  |  |
| 50 GT  Family  (4,866) | GT2 | 1289 | GT26 | 243 | GT30 | 90 | GT3 | 41 | GT10 | 13 | GT29 | 8 | GT41 | 3 | GT90 | 1 | GT81 | 1 |
|  | GT4 | 575 | GT8 | 195 | GT84 | 75 | GT83 | 38 | GT100 | 13 | GT17 | 7 | GT13 | 3 | GT21 | 1 | GT92 | 1 |
|  | GT51 | 388 | GT66 | 141 | GT22 | 73 | GT11 | 30 | GT96 | 11 | GT20 | 6 | GT42 | 3 | GT102 | 1 |  |  |
|  | GT35 | 365 | GT9 | 134 | GT1 | 67 | GT14 | 26 | GT99 | 10 | GT25 | 4 | GT6 | 3 | GT103 | 1 |  |  |
|  | GT28 | 347 | GT47 | 113 | GT19 | 48 | GT101 | 19 | GT104 | 9 | GT74 | 4 | GT34 | 2 | GT56 | 1 |  |  |
|  | GT5 | 291 | GT32 | 100 | GT39 | 42 | GT87 | 15 | GT82 | 9 | GT23 | 3 | GT7 | 2 | GT57 | 1 |  |  |
| 16 PL  Family  (220) | PL1 | 55 | PL8 | 32 | PL12 | 16 | PL26 | 14 | PL21 | 4 | PL17 | 3 | PL15 | 2 | PL16 | 1 |  |  |
|  | PL11 | 46 | PL10 | 18 | PL9 | 15 | PL22 | 5 | PL27 | 4 | PL6 | 3 | PL13 | 1 | PL4 | 1 |  |  |
| 13 CE  Family  (1,166) | CE4 | 356 | CE12 | 164 | CE1 | 82 | CE14 | 43 | CE2 | 34 | CE6 | 22 | CE16 | 3 |  |  |  |  |
|  | CE9 | 217 | CE11 | 95 | CE8 | 60 | CE15 | 42 | CE7 | 32 | CE3 | 16 |  |  |  |  |  |  |
| AA Family  (8) | AA10 | 8 |  |  |  |  |  |  |  |  |  |  |  |  |  |  |  |  |
| 54 CBM  Family (1,935) | CBM50 | 420 | CBM2 | 65 | CBM4 | 29 | CBM9 | 19 | CBM27 | 9 | CBM3 | 6 | CBM58 | 4 | CBM42 | 2 | CBM59 | 1 |
|  | CBM48 | 335 | CBM35 | 64 | CBM5 | 28 | CBM57 | 18 | CBM25 | 9 | CBM40 | 5 | CBM10 | 4 | CBM30 | 2 | CBM43 | 1 |
|  | CBM32 | 158 | CBM51 | 56 | CBM37 | 26 | CBM61 | 15 | CBM47 | 7 | CBM36 | 5 | CBM16 | 3 | CBM79 | 1 | CBM38 | 1 |
|  | CBM13 | 155 | CBM20 | 50 | CBM26 | 26 | CBM66 | 14 | CBM23 | 7 | CBM77 | 4 | CBM80 | 2 | CBM72 | 1 | CBM18 | 1 |
|  | CBM34 | 137 | CBM41 | 39 | CBM62 | 25 | CBM22 | 14 | CBM82 | 6 | CBM68 | 4 | CBM75 | 2 | CBM70 | 1 | CBM11 | 1 |
|  | CBM6 | 76 | CBM67 | 31 | CBM54 | 21 | CBM12 | 11 | CBM56 | 6 | CBM65 | 4 | CBM74 | 2 | CBM69 | 1 | CBM1 | 1 |

**Table S5** Distribution of actinomycetes isolated from the faecal samples of *R. bieti* at different classification levels.

| Order | Family | Genus | Strain No. |
| --- | --- | --- | --- |
| Cellulomonadales | Cellulomonadaceae | *Cellulomonas* | 2 |
|  | Jonesiaceae | *Sanguibacter* | 1 |
|  | Oerskoviaceae | *Oerskovia* | 15 |
| Dermabacterales | Dermabacteraceae | *Brachybacterium* | 1 |
| Microbacteriales | Microbacteriaceae | *Agrococcus* | 1 |
|  |  | *Curtobacterium* | 4 |
|  |  | *Microbacterium* | 16 |
|  |  | *Mycetocola* | 2 |
|  |  | *Plantibacter* | 6 |
|  |  | *Pseudoclavibacter* | 2 |
| Micrococcales | Micrococcaceae | *Arthrobacter* | 97 |
|  |  | *Glutamicibacter* | 2 |
|  |  | *Kocuria* | 3 |
|  |  | *Micrococcus* | 2 |
|  |  | *Paenarthrobacter* | 1 |
|  |  | *Paeniglutamicibacter* | 1 |
| Micromonosporales | Micromonosporaceae | *Micromonospora* | 1 |
| Mycobacteriales | Corynebacteriaceae | *Corynebacterium* | 1 |
|  | Dietziaceae | *Dietzia* | 1 |
|  | Gordoniaceae | *Gordonia* | 4 |
|  |  | *Williamsia* | 16 |
|  | Nocardiaceae | *Rhodococcus* | 17 |
| Propionibacteriales | Nocardioidaceae | *Aeromicrobium* | 1 |
|  | Propionibacteriaceae | *Luteococcus* | 1 |
| Streptomycetales | Streptomycetaceae | *Streptomyces* | 23 |

**Table S6** Distribution of other bacteria isolated from the faecal samples of *R. bieti* at different classification levels.

| Phylum | Class | Order | Family | Genus | Strain No. |
| --- | --- | --- | --- | --- | --- |
| Bacteroidetes | Flavobacteriia | Flavobacteriales | Flavobacteriaceae | *Faecalibacter* | 1 |
|  |  |  |  | *Flavobacterium* | 1 |
|  | Sphingobacteriia | Sphingobacteriales | Sphingobacteriaceae | *Pedobacter* | 2 |
|  |  |  |  | *Sphingobacterium* | 25 |
| Deinococcus-Thermus | Deinococci | Deinococcales | Deinococcaceae | *Deinococcus* | 1 |
| Proteobacteria | Alphaproteobacteria | Caulobacterales | Caulobacteraceae | *Brevundimonas* | 3 |
|  | Betaproteobacteria | Burkholderiales | Comamonadaceae | *Acidovorax* | 1 |
|  |  |  |  | *Comamonas* | 21 |
|  | Gammaproteobacteria | Enterobacterales | Budviciaceae | *Budvicia* | 1 |
|  |  |  | Enterobacteriaceae | *Escherichia* | 4 |
|  |  |  |  | *Lelliottia* | 1 |
|  |  |  |  | *Raoultella* | 1 |
|  |  |  | Morganellaceae | *Providencia* | 1 |
|  |  |  | Yersiniaceae | *Rahnella* | 7 |
|  |  |  |  | *Rouxiella* | 1 |
|  |  |  |  | *Serratia* | 8 |
|  |  |  |  | *Yersinia* | 15 |
|  |  | Lysobacterales | Lysobacteraceae | *Stenotrophomonas* | 6 |
|  |  | Pseudomonadales | Moraxellaceae | *Acinetobacter* | 1 |
|  |  |  |  | *Prolinoborus* | 2 |
|  |  |  | Pseudomonadaceae | *Pseudomonas* | 21 |
| Firmicutes | Bacilli | Bacillales | Bacillaceae | *Bacillus* | 10 |
|  |  |  |  | *Peribacillus* | 2 |
|  |  |  | Paenibacillaceae | *Paenibacillus* | 2 |
|  |  |  | Planococcaceae | *Planococcus* | 1 |
|  |  |  |  | *Psychrobacillus* | 13 |
|  |  |  | Staphylococcaceae | *Staphylococcus* | 1 |
|  |  | Lactobacillales | Carnobacteriaceae | *Carnobacterium* | 19 |
|  |  |  | Enterococcaceae | *Enterococcus* | 18 |
|  |  |  | Leuconostocaceae | *Leuconostoc* | 1 |

**Table S7** Potential new bacterial taxa isolated from the faeces of *R. bieti*.

| Strain | Most similar strain | Identity（%） |
| --- | --- | --- |
| WQ 2009 | *Sphingobacterium kitahiroshimense* | 94.5 |
| WQ 047 | *Sphingobacterium nematocida* | 96.4 |
| WQ 075 | *Planomicrobium flavidum* | 96.7 |
| WQ 2020 | *Mycetocola reblochoni* | 96.8 |
| WQ 111 | *Acinetobacter gandensis* | 96.9 |
| WQ 154 | *Arthrobacter livingstonensis* | 97.2 |
| WQ 166 | *Pedobacter bauzanensis* | 97.5 |
| WQ 117 | *Faecalibacter macacae* | 97.5 |
| WQ 122 | *Curtobacterium flaccumfaciens* | 97.6 |
| WQ 308 | *Microbacterium hatanonis* | 98.0 |
| WQ 314 | *Comamonas jiangduensis* | 98.3 |
| WQ 920 | *Acidovorax radicis* | 98.4 |
| WQ 090 | *Budvicia aquatica* | 98.4 |
| WQ 081 | *Cellulomonas cellasea* | 98.5 |
| WQ 033 | *Prolinoborus fasciculus* | 98.5 |
| WQ 2005 | *Arthrobacter koreensis* | 98.6 |
| WQ 186 | *Arthrobacter luteolus* | 98.6 |
| WQ 005 | *Paenibacillus macquariensis subsp. macquariensis* | 98.6 |
| WQ 310 | *Rhodococcus coprophilus* | 98.7 |
| WQ 095 | *Plantibacter flavus* | 98.7 |

**Table S8** CAZymes predicted in the type species from all genera of family *Sphingobacteriaceae*.

| Strains | Genome size (bp) | CAZyme No. | | | | | | |
| --- | --- | --- | --- | --- | --- | --- | --- | --- |
|  |  | Total | GH | GT | CBM | CE | AA | PL |
| WQ 2009^T^ | 3,144,451 | 166 | 80 | 37 | 30 | 14 | 5 | - |
| *Sphingobacterium kitahiroshimense* 10C^T^ | 6,247,893 | 336 | 173 | 68 | 56 | 25 | 7 | 7 |
| *Sphingobacterium pakistanense* NCCP-246^T^ | 5,842,415 | 328 | 169 | 70 | 56 | 19 | 7 | 7 |
| *Sphingobacterium faecium DSM* 11690^T^ | 5,296,794 | 303 | 148 | 77 | 37 | 31 | 6 | 4 |
| *Sphingobacterium detergens* CECT 7938^T^ | 6,733,110 | 354 | 186 | 69 | 58 | 24 | 9 | 8 |
| *Sphingobacterium siyangense* CGMCC 1.6855^T^ | 6,294,929 | 369 | 211 | 60 | 52 | 32 | 5 | 9 |
| *Sphingobacterium spiritivorum* NCTC 11386^T^ | 5,138,967 | 245 | 116 | 50 | 37 | 26 | 7 | 9 |
| *Albibacterium bauzanense* BZ42^T^ | 3,081,443 | 194 | 80 | 58 | 30 | 23 | 3 | - |
| *Daejeonella oryzae* DSM 19973^T^ | 3,445,768 | 182 | 61 | 80 | 20 | 16 | 3 | 2 |
| *Arcticibacter svalbardensis* MN12-7^T^ | 4,687,695 | 350 | 169 | 115 | 25 | 18 | 2 | 21 |
| *Pararcticibacter amylolyticus* FJ4-8^T^ | 6,314,794 | 495 | 277 | 93 | 68 | 40 | 2 | 15 |
| *Nubsella zeaxanthinifaciens* TDMA-5^T^ | 4,252,742 | 240 | 88 | 83 | 24 | 27 | 4 | 14 |
| *Pelobium manganitolerans* YS-25^T^ | 3,932,770 | 360 | 170 | 83 | 39 | 35 | 7 | 26 |
| *Mucilaginibacter agri* R11^T^ | 5,607,981 | 352 | 164 | 105 | 37 | 28 | 11 | 7 |
| *Mucilaginibacter paludis* TPT56^T^ | 8,408,322 | 488 | 266 | 93 | 65 | 44 | 3 | 17 |
| *Anseongella ginsenosidimutans* DSM 21100^T^ | 4,252,511 | 305 | 135 | 82 | 57 | 21 | 5 | 5 |
| *Olivibacter sitiensis* DSM 17696^T^ | 5,053,571 | 319 | 172 | 83 | 37 | 19 | 7 | 1 |
| *Pseudopedobacter saltans* DSM 12145^T^ | 4,635,236 | 438 | 215 | 91 | 40 | 40 | 5 | 48 |
| *Solitalea koreensis* DSM 21342^T^ | 3,342,189 | 187 | 91 | 53 | 29 | 12 | 2 | - |
| *Parapedobacter indicus* RK1^T^ | 6,155,666 | 414 | 222 | 92 | 48 | 39 | 6 | 7 |
